# Pilot study indicate role of preferentially transmitted monoamine oxidase gene variants in behavioral problems of male ADHD probands

**DOI:** 10.1186/s12881-017-0469-5

**Published:** 2017-10-05

**Authors:** Arijit Karmakar, Rishov Goswami, Tanusree Saha, Subhamita Maitra, Anirban Roychowdhury, Chinmay Kumar Panda, Swagata Sinha, Anirban Ray, Kochupurackal P. Mohanakumar, Usha Rajamma, Kanchan Mukhopadhyay

**Affiliations:** 1 Manovikas Biomedical Research and Diagnostic Centre, 482, Madudah, Plot: I-24, Sector-J, Manovikas Kendra, E.M. Bypass, Kolkata, 700 107 India; 2 Department of Oncogene Regulation, Chittaranjan National Cancer Institute, 37 S.P. Mukherjee Road, Kolkata, 700 026 India; 3 Department of Psychiatry, Institute of Post Graduate Medical Education & Research, Kolkata, 700 020 India; 4 CSIR-Indian Institute of Chemical Biology, Laboratory of Clinical & Experimental Neurosciences, Cell Biology & Physiology Division, 4, Raja S.C. Mullick Road, Kolkata, 700 032 India; 5 Inter University Centre for Biomedical Research & Super Specialty Hospital, Mahatma Gandhi University Campus at Thalappady, Rubber Board PO, Kottayam, 686 009 Kerala State India

**Keywords:** ADHD, *MAOA*, *MAOB*, Genotyping, Maternal transmission, Behavioral trait, Maternal age, Linkage disequilibrium, Indo-Caucasoid population

## Abstract

**Background:**

Attention deficit hyperactivity disorder (ADHD) is an etiologically complex childhood onset neurobehavioral disorder characterized by age-inappropriate inattention, hyperactivity, and impulsivity. Symptom severity varies widely and boys are diagnosed more frequently than girls. ADHD probands were reported to have abnormal transmissions of dopamine, serotonin, and/or noradrenaline. Monoamine oxidase A (MAOA) and B (MAOB), mitochondrial outer membrane bound two isoenzymes, mediate degradation of these neurotransmitters and thus regulating their circulating levels. Case-control analyses in different populations, including Indians, suggested involvement of *MAOA* and *MAOB* genes in the etiology of ADHD. Due to high heritability rate of ADHD, we tested familial transmission of *MAOA* and *MAOB* variants to ADHD probands in 190 nuclear families having ADHD probands from Indo-Caucasoid ethnicity.

**Methods:**

Subjects were recruited following the Diagnostic and Statistical Manual of Mental Disorders-4th edition (DSM-IV). Appropriate scales were used for measuring the behavioral traits in probands. Genotyping was performed through PCR-based amplification of target sites followed by DNA-sequencing and/or gel-electrophoresis. Data obtained were analyzed by family based statistical methods.

**Results:**

Out of 58 variants present in the analyzed sites only 15 were found to be polymorphic (30 bp-uVNTR, rs5906883, rs1465107, rs1465108, rs5905809, rs5906957, rs6323, rs1137070 from *MAOA* and rs4824562, rs56220155, rs2283728, rs2283727, rs3027441, rs6324, rs3027440 from *MAOB*). Statistically significant maternal transmission of alleles to male probands was observed for *MAOA* rs5905809 ‘*G*’ (*p* = 0.04), rs5906957 ‘*A*’ (*p* = 0.04), rs6323 ‘*G*’ (*p* = 0.0001) and *MAOB* rs56220155 ‘*A*’ (*p* = 0.002), rs2283728 ‘*C*’ (*p* = 0.0008), rs2283727 ‘*C*’ (*p* = 0.0008), rs3027441 ‘*T*’ (*p* = 0.003), rs6324 ‘*C*’ (*p* = 0.003), rs3027440 ‘*T*’ (*p* = 0.0002). Significantly preferential maternal transmissions of different haplotype combinations to male probands were also noticed (*p* < 0.05), while female probands did not reveal such transmission bias. Behavioral traits of male probands exhibited significant association with gene variants. Age of the mother at pregnancy also revealed association with risk variants of male probands.

**Conclusions:**

It may be inferred that the *MAOA* and *MAOB* variants may contribute to the etiology of ADHD in the Indo-Caucasoid population and could be responsible for higher occurrence of ADHD in the boys.

**Electronic supplementary material:**

The online version of this article (10.1186/s12881-017-0469-5) contains supplementary material, which is available to authorized users.

## Background

Attention deficit hyperactivity disorder (ADHD) is an etiologically complex neurobehavioral disorder, diagnosed mostly during early childhood [1]. The disorder is highly prevalent throughout the world, including India, and boys are more frequently diagnosed with ADHD as compared to girls [2–6]. Age-inappropriate persistent and pervasive symptoms of inattention, hyperactivity, and impulsivity [1] often lead to impairments in academic performances as well as social life [7, 8]. Other psychiatric conditions, frequently detected as co-morbidity in subjects with ADHD, may make the situation worse [9, 10]. Thus an early diagnosis, leading to early intervention, becomes crucial for successful management.

Being a multi-factorial disorder with almost 76% heritability [11, 12], ADHD is believed to have significant influence of multiple gene variants [13–16]. Candidate genes involved in the regulation of dopamine, serotonin, and noradrenalin were widely studied in ADHD subjects since behavioral traits are regulated by these neurotransmitters [13, 15, 17, 18] and both dopaminergic [15, 19–21] and serotonergic [22] transmissions revealed significant impact on behavioral as well as cognitive features.

Two flavin containing isoenzymes, monoamine oxidase A (MAOA) and B (MAOB), help in the deamination of biogenic amines from both endogenous and exogenous sources [23, 24]. Both the enzymes are localized in the mitochondrial outer membrane and metabolize dopamine, tyramine, and tryptamine with equal efficiency [25–27]. Comparative analysis also revealed that while MAOA preferentially deaminates serotonin, noradrenalin, adrenaline, and melatonin, preferred substrates for MAOB are phenylethylamine (PEA) and benzylamine [25–27].

In men of a Dutch family, MAOA deficiency showed association with aggressive behavior [28] and deletion of the *MAOA* gene showed association with aggressive phenotypes across species [29–31]. MAOA also exhibited association with vulnerability to disorders of attention and impulsivity [32] and a possible link between predisposition to novelty seeking [33], making the *MAOA* gene, encoding for the MAOA enzyme, a prime candidate for ADHD [31, 32, 34, 35].

MAOB was also postulated to regulate impulsivity, attention and vulnerability to ADHD through metabolism of dopamine, although to a lesser extent as compared to MAOA [20, 32]. Correlation of platelet MAOB activity with sensation seeking and other behavioral abnormalities have also been reported [36, 37]. Negative emotionality of healthy volunteers showed association with *MAOB* polymorphisms [38]. However, platelet MAOB activity failed to correlate with brain MAOB activity, thus questioning the usefulness of MAOB as a marker for psychiatric behavior [39].

Proportional analysis in patients revealed that the absence of MAOA leads to greater change in neurotransmitter metabolism than absence of MAOB [40]. While *MAOA* knockout mice showed increased levels of serotonin, norepinephrine and dopamine in the brain [41], only level of PEA was increased in *MAOB* knockout mice [42]. MAOA and MAOB double knockout mice showed an increased reactivity to stress [30] and increased levels of serotonin, norepinephrine, dopamine, and PEA in the brain to a much greater degree than in either *MAOA* or *MAOB* single knockout mice [43].

In this backdrop of information, both *MAOA* and *MAOB* genes, located on the X chromosome [26], were considered to contribute to the etiology of ADHD [31, 32, 34, 44–46]. However, worldwide only a few *MAOA* and *MAOB* gene variants were studied and the data obtained were neither consistent nor conclusive [31, 35, 37, 44–47]. Our population-based analysis on 58 *MAO* gene variants revealed association of a number of variants with ADHD [48, 49]. Due to high heritability of ADHD traits, in the present study all these variants were explored to identify familial transmission pattern. Additionally, based on the X-chromosomal location of *MAOA* and *MAOB* [46], we have performed gender based stratified analysis to identify whether any variant is preferentially transmitted to the probands and thus may have a role in the gender bias often reported in ADHD.

## Methods

### Subject recruitment

A total of 190 ADHD probands (166 males and 24 females) and their biological parents, of Indo-Caucasoid ethnicity from the eastern India, were recruited from the out-patient department of Manovikas Kendra Rehabilitation and Research Institute for the Handicapped, Kolkata, India. Diagnosis was performed by child psychiatrist and clinical psychologist following the Diagnostic and Statistical Manual of Mental Disorders-4th edition (DSM-IV) criteria [50]. 74.74% of the recruited ADHD probands were of the combined subtype, while inattentive and hyperactive-impulsive subtypes were of 13.68% and 11.58% respectively. Mean age of the ADHD probands was 8.01 ± 0.22 years (Mean ± SE). Psychological evaluation was done through – the revised Conners’ Parents Rating Scale (CPRS-R) [51] and Wechsler Intelligence Scale for Children >5 yrs. [52] / Developmental Screening Test [53] for children <5 yrs. for the inattention-hyperactivity level and intelligent quotient (IQ)/ developmental quotient (DQ) status respectively. Oppositional defiant disorder (ODD) and conduct problems of ADHD probands were assessed using the DSM-IV score and Parental Account of Children’s Symptoms (PACS) score respectively. Probands with any other neuropsychiatric disorders, mental retardation (IQ ≤ 70) including Down syndrome and Fragile-X syndrome, pervasive developmental disorder were excluded from the study. Among 166 male ADHD probands 133 were trios, 25 were duos (6 excluding mother and 19 excluding father) and 8 were single probands. Among the 24 female probands, 15 were trios, 6 were duos (having mother only) and 3 were single probands. Informed written consent was obtained from guardians / biological parents of the probands participating in the study and the protocol was approved by the Institutional Human Ethical Committee.

### Genotyping

Peripheral blood of the study participants was collected by a trained phlebotomist and used for genomic DNA preparation following the standard protocol [54]. The target regions were amplified via polymerase chain reaction (PCR) using oligonucleotides designed in the lab using the Primer3 software [55] and the amplicons were utilized for genotyping the samples either through gel electrophoresis or using Sanger sequencing by capillary electrophoresis method [56]. For sequence analysis of the amplicons, Applied Biosystems 3130 Genetic Analyzer was used [48, 49]. Chromatograms were also analyzed manually and for the identification of heterozygous SNPs, >25% base calling was accepted. Detailed analytic protocols for PCR amplification and genotyping were published earlier [48, 49].

### Statistical analyses of data

Genotypic counts of only the female subjects (i.e. female ADHD probands and mother of the probands) were used for analyzing the Hardy-Weinberg equilibrium [57], since the *MAO* genes are located on the X-chromosome. For the same reason, family-based analysis on male ADHD probands was carried out considering only the maternal transmission. Paternal transmission was considered for female ADHD probands only. Haplotype-based haplotype relative risk (HHRR) analysis [58] was performed using UNPHASED v 3.1.5 [59] to identify allelic and haplotypic transmission patterns. Correction for multiple testing was done while running the UNPHASED at 1000-fold iteration. Relative risk or Risk ratio for variants showing significant association was calculated online [60]. Pair-wise linkage disequilibrium (LD) between the variants was calculated using the Haploview program version 4.2 [61], considering male and female probands separately. Since *MAO* genes are X-linked, for female probands the parental genotype data and for male probands only the maternal genotype data were used for comparative analysis.

### Stratified analysis on behavioral traits, age-of-onset, and maternal age at pregnancy

Based on the CPRS-R, respective ‘T scores’ for oppositional behavior, cognitive problems / inattention, hyperactivity, and ADHD index were obtained for ADHD probands (*N* = 166). DSM-IV score for ODD trait and PACS for conduct problems of ADHD probands were also obtained. CPRS-R ‘T scores’ ranged between 38 to 90 while DSM-IV scores and PACS scores ranged between 0 to 36 and 0 to 90 respectively. Behavioral traits/scores of ADHD probands were utilized for genetic association analysis. Male probands were divided into two sub-groups based on the presence/absence of the derived allele of each variant. Allelic association with behavioral scores was analyzed using Student’s t-test [62] in the presence of normal distribution of data and equal variances in the two comparing groups. Age of the male ADHD probands at the time of onset of the disorder were used for stratified analysis; probands with detectable symptoms at an age ≤ 7 years (*N* = 109) were considered under ‘early onset’, while those with detectable symptoms after 7 years (*N* = 57) were classified under the ‘late onset’. Association of alleles with age-of-onset of ADHD in the male probands was calculated using the chi-square test [62]. To calculate the impact of maternal age at pregnancy, allelic frequencies of male probands born to mothers ≤26 years (*N* = 71) were compared to that of male probands born to mothers at >26 years (*N* = 81) of age using the chi-square test [62]. As the number of female probands was limited, association of variants with behavioral scores, age-of-onset of ADHD, and maternal age at pregnancy were not analyzed.

## Results

Out of 58 variants present in the analyzed sites, only 15 (30 bp-uVNTR, rs5906883, rs1465107, rs1465108, rs5905809, rs5906957, rs6323, rs1137070 from *MAOA* and rs4824562, rs56220155, rs2283728, rs2283727, rs3027441, rs6324, rs3027440 from *MAOB*) were found to be polymorphic. All the studied polymorphic variants followed the Hardy-Weinberg equilibrium in the female subjects, i.e. female ADHD probands and mother of the ADHD probands (Additional file 1). Family-based analysis showed statistically significant maternal over-transmission of *MAOA* rs5905809 ‘*G*’ (*p* = 0.04), rs5906957 ‘*A*’ (*p* = 0.04) and rs6323 ‘*G*’ (*p* = 0.0001) alleles and *MAOB* rs56220155 ‘*A*’ (*p* = 0.002), rs2283728 ‘*C*’ (*p* = 0.0008), rs2283727 ‘*C*’ (*p* = 0.0008), rs3027441 ‘*T*’ (*p* = 0.003), rs6324 ‘*C*’ (*p* = 0.003) and rs3027440 ‘*T*’ (*p* = 0.0002) alleles to the male probands (Table 1). The relative risk was also statistically significant for *MAOA* rs5905809 ‘*G*’, rs5906957 ‘*A*’ and rs6323 ‘*G*’ and all the *MAOB* variants excepting for rs4824562 (Table 1). No such parental bias in transmissions was observed in the female probands (Additional file 2). Several intra-genetic as well as inter-genetic haplotypic combinations also showed statistically significant maternal over-transmission to the male probands (*p* ≤ 0.05) (Table 2 & Additional file 3). Most significantly over-transmitted (*p* = 0.002) intra-genetic haplotypes of *MAOA* were rs5906883-rs6323 ‘*C-G*’, rs5905809-rs6323 ‘*G-G*’, and rs5906957-rs6323 ‘*A-G*’ (Table 2). Most significantly over-transmitted (*p* = 1.66E-05) intra-genetic haplotypes of *MAOB* were rs3027440-rs2283727 ‘*T-C*’ and rs3027440-rs2283728 ‘*T-C*’ (Table 2). Inter-genetic haplotype combinations, between *MAOA* and *MAOB*, showing over-transmission (*p* = 2.05E-05) were rs6323-rs2283727 ‘*G-C*’ and rs6323-rs2283728 ‘*G-C*’ (Table 2). Preferential non-transmission (*p* ≤ 0.05) of several haplotypes were also noticed (Additional file 4) in the male probands. No such significant biased parental transmissions of haplotypes were observed in the female probands (Additional file 5).

**Table 1:** Maternal allelic transmission to male ADHD probands

Genes	Variants	Alleles	Transmitted	Non-transmitted	Chi-square (*p*-value)	Relative risk (95% confidence interval)
*MAOA*	30 bp-uVNTR	*3R*	0.68	0.65	0.37 (0.54)	–
		*4R*	0.32	0.35		
	rs5906883	*A*	0.67	0.64	0.36 (0.55)	–
		*C*	0.33	0.36		
	rs1465107	*G*	0.31	0.39	2.44 (0.12)	–
		*A*	0.69	0.61		
	rs1465108	*A*	0.69	0.61	2.44 (0.12)	–
		*G*	0.31	0.39		
	rs5905809	*C*	0.33	0.44	4.03 (**0.04**)	1.20 (1.00 – 1.44)
		*G*	0.67	0.56		
	rs5906957	*A*	0.67	0.56	4.03 (**0.04**)	1.20 (1.00 – 1.44)
		*G*	0.33	0.44		
	rs6323	*T*	0.24	0.45	15.15 (**0.0001**)	1.38 (1.17 – 1.63)
		*G*	0.76	0.55		
	rs1137070	*C*	0.32	0.41	2.40 (0.12)	–
		*T*	0.68	0.59		
*MAOB*	rs4824562	*A*	0.80	0.73	2.23 (0.14)	–
		*G*	0.20	0.27		
	rs56220155	*G*	0.26	0.43	9.16 (**0.002**)	1.29 (1.09 – 1.52)
		*A*	0.74	0.57		
	rs2283728	*T*	0.19	0.36	11.26 (**0.0008**)	1.27 (1.10 – 1.46)
		*C*	0.81	0.64		
	rs2283727	*C*	0.81	0.64	11.26 (**0.0008**)	1.27 (1.10 – 1.46)
		*A*	0.19	0.36		
	rs3027441	*C*	0.20	0.35	8.86 (**0.003**)	1.23 (1.07 – 1.42)
		*T*	0.80	0.65		
	rs6324	*C*	0.80	0.65	8.86 (**0.003**)	1.23 (1.07 – 1.42)
		*T*	0.20	0.35		
	rs3027440	*T*	0.86	0.68	13.67 (**0.0002**)	1.26 (1.11 – 1.43)
		*C*	0.14	0.32		

Statistically significant differences are presented in bold

**Table 2:** Intra- and inter-genetic haplotypes transmitted most significantly to male ADHD probands from mothers

Combinations	Variants	Haplotype	Transmitted	Non-transmitted	Chi-square (*p*-value)
Intra genetic in *MAOA*	rs5906883-rs6323	*C-G*	0.15	0.05	9.47 (**0.002**)
	rs5905809-rs6323	*G-G*	0.62	0.44	9.63 (**0.002**)
	rs5906957-rs6323	*A-G*	0.62	0.44	9.63 (**0.002**)
Inter genetic between *MAOA* and *MAOB*	rs6323-rs2283727	*G-C*	0.58	0.34	18.15 (**2.05E-05**)
	rs6323-rs2283728	*G-C*	0.58	0.34	18.15 (**2.05E-05**)
Intra genetic in *MAOB*	rs3027440-rs2283727	*T-C*	0.80	0.57	18.55 (**1.66E-05**)
	rs3027440-rs2283728	*T-C*	0.80	0.57	18.55 (**1.66E-05**)

Statistically significant differences are presented in bold

**Fig. 1:**
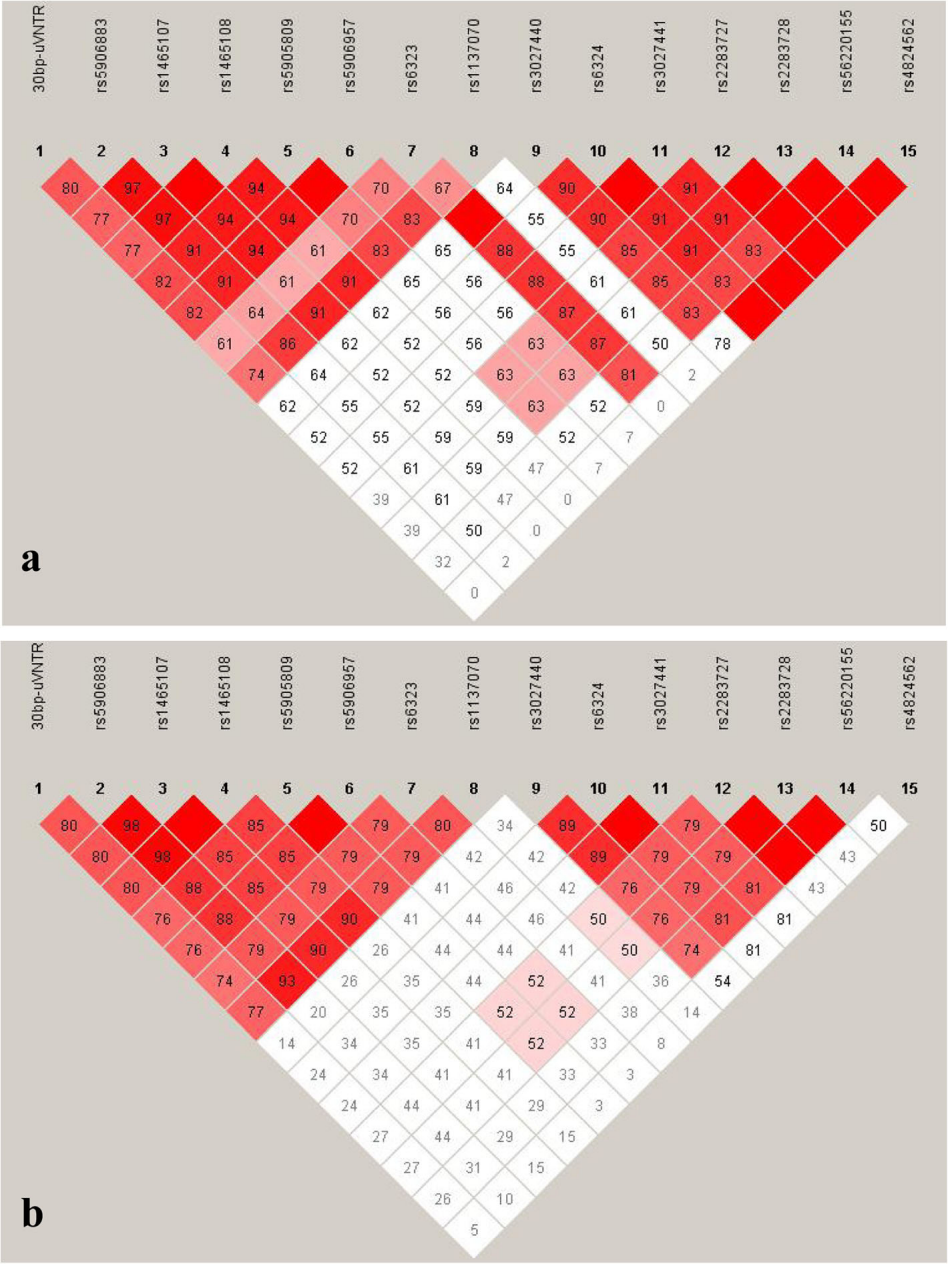
Plot of pair-wise linkage disequilibrium (LD) between the polymorphic variants from *MAOA* and *MAOB* genes in **a**. Male ADHD cases; **b**. Mothers of the male ADHD cases. Diamonds without numbers represent *D’* values of 1.0; all numbers represent the *D’* value expressed as a percentile. *D’* is a measure of the frequency of association of alleles at 2 loci

**Fig. 2:**
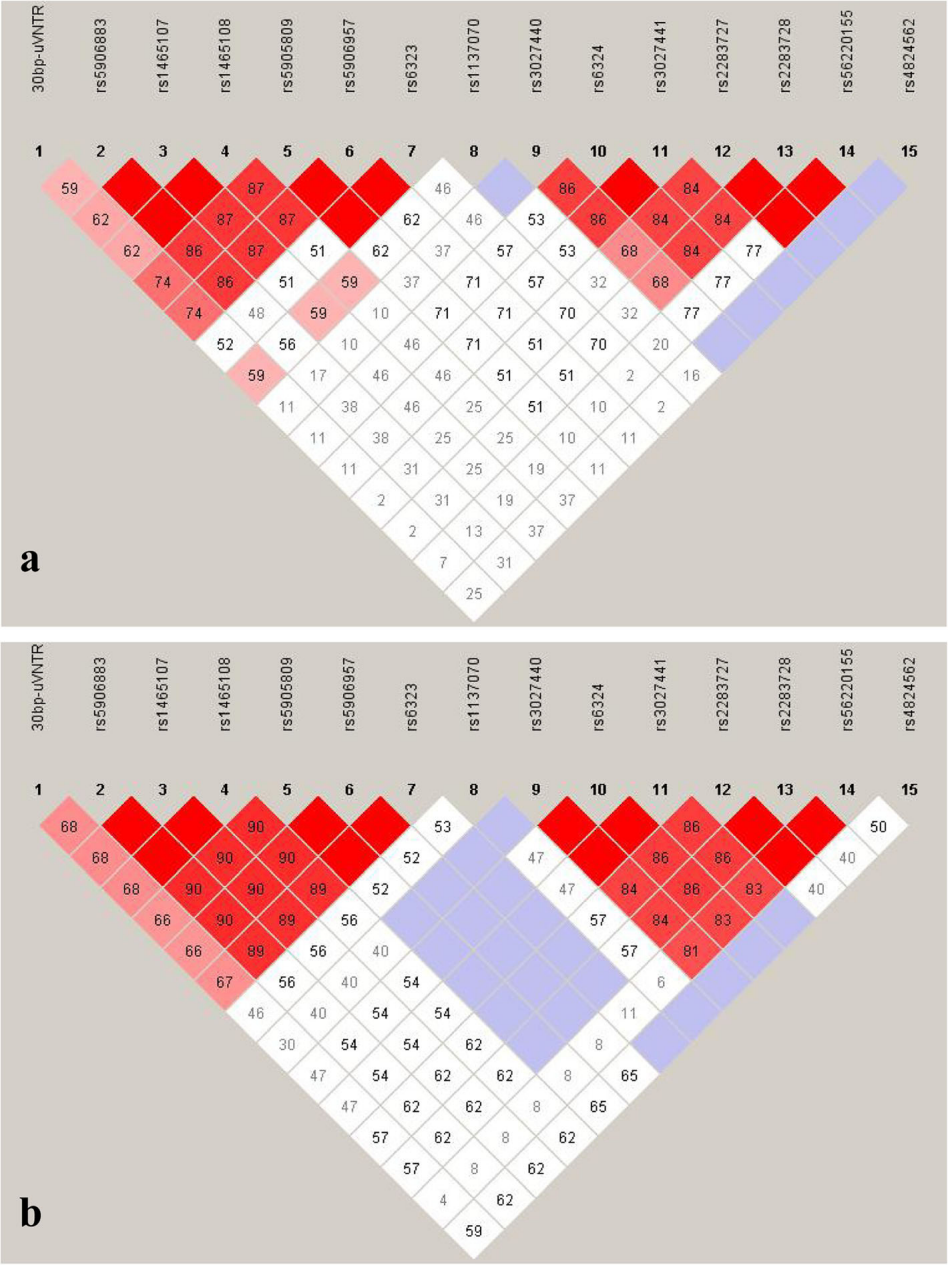
Plot of pair-wise linkage disequilibrium (LD) between the polymorphic variants from *MAOA* and *MAOB* genes in **a**. Female ADHD cases; **b**. Parents of the female ADHD cases. Diamonds without numbers represent *D’* values of 1.0; all numbers represent the *D’* value expressed as a percentile. *D’* is a measure of the frequency of association of alleles at 2 loci

In the male ADHD probands, intra-genetic pair-wise LDs for *MAOA* and *MAOB* variants were found to be same as reported earlier [48, 49]. Inter-genetic pair-wise analysis revealed strong LD of *MAOA* rs6323 with *MAOB* rs3027440, rs6324, rs3027441, rs2283727, rs2283728, and rs56220155 in the male probands (Fig. 1a, Additional file 6). In mothers of the male probands, all the variants from *MAOA* gene were found to be in pair-wise LDs with each other (Fig. 1b, Additional file 6). Complete pair-wise LDs were found between rs1465107 and rs1465108; and rs5905809 and rs5906957 respectively (Fig. 1b, Additional file 6). In the *MAOB*, all the variants, except rs4824562, were found to be in strong pair-wise LDs with each other (Fig. 1b, Additional file 6). Complete pair-wise LDs were found between rs6324 and rs3027441; and rs2283727 and rs2283728 respectively (Fig. 1b, Additional file 7). Strong pair-wise LDs of rs56220155 with rs2283727 and rs2283728 respectively were also observed (Fig. 1b, Additional file 6). In the female ADHD probands, no inter-genetic pair-wise LDs were observed (Fig. 2a, Additional file 6). Intra-genetic pair-wise LDs were found to be same as reported earlier [48, 49]. In the group of parents of the female probands all the variants from *MAOA* gene, except 30 bp-uVNTR and rs1137070, were found to be in pair-wise LDs with each other (Fig. 2b, Additional file 6). Complete pair-wise LDs were found between rs1465107 and rs1465108; and rs5905809 and rs5906957 respectively (Fig. 2b, Additional file 6). Strong pair-wise LDs of rs5906883 with rs1465107 and rs1465108 respectively were observed (Fig. 2b, Additional file 6). Strong pair-wise LDs of rs6323 with rs5905809 and rs5906957 respectively were also observed (Fig. 2b, Additional file 6). From *MAOB* gene, all the variants, except rs4824562, were found to be in strong pair-wise LDs with each other (Fig. 2b, Additional file 6). Complete pair-wise LDs were found between rs6324 and rs3027441; and rs2283727 and rs2283728 respectively (Fig. 2b, Additional file 6). Strong pair-wise LDs of rs3027440 with rs6324 and rs3027441 respectively were observed (Fig. 2b, Additional file 6). Strong pair-wise LDs of rs56220155 with rs2283727 and rs2283728 respectively were also noticed (Fig. 2b, Additional file 6).

Behavioral traits were calculated by obtaining Mean ± standard error (SE). CPRS-R ‘T scores’ obtained were 62.81 ± 1.23 for oppositional behavior, 72.38 ± 0.79 for cognitive problems / inattention, 74.50 ± 0.99 for hyperactivity, and 71.74 ± 0.68 for ADHD index. DSM-IV scores for ODD trait was 15.05 ± 0.85 and PACS scores for conduct problems was 16.77 ± 1.19. Male ADHD probands having rs6323 ‘*G*’ allele showed statistically significant higher mean ‘T score’ for hyperactivity and ADHD index as compared to those with the ‘*T*’ allele (Table 3). Significantly higher mean ‘DSM-IV score’ for ODD trait was also noticed in the male ADHD probands having rs1137070 ‘*C*’ allele as compared to the male probands having the ‘*T*’ allele (Table 3). *MAOB* rs2283728 ‘*T*’ and rs2283727 ‘*A*’ showed statistically significant association with higher mean ‘T score’ for hyperactivity in the male ADHD probands in comparison to probands having the ‘*C*’ alleles of the respective variants (Table 3). Significantly higher mean ‘T score’ for cognitive problems / inattention and ADHD index were also noticed in the male ADHD probands having rs3027441 ‘*C*’ and rs6324 ‘*T*’ alleles than the male ADHD probands having ‘*T*’ and ‘*C*’ alleles of the respective variants (Table 3). No significant association between ‘T scores’ for oppositional behavior and PACS scores for conduct problems were noticed (Additional file 7). Age of onset of ADHD in male probands also failed to show any significant association (Additional file 8).

**Table 3:** Analysis of association between alleles & ADHD associated trait scores of male ADHD probands

Genes	Variants	Alleles	‘T scores’ for cognitive problems / inattention		‘T scores’ for hyperactivity		‘T scores’ for ADHD index		DSM-IV scores for ODD trait	
			Mean ± SE	*p*	Mean ± SE	*p*	Mean ± SE	*p*	Mean ± SE	*p*
*MAOA*	30 bp-uVNTR	*3R*	71.90 ± 1.03	0.26	75.16 ± 1.23	0.15	71.27 ± 0.83	0.29	14.75 ± 1.10	0.32
		*4R*	70.77 ± 1.37		72.93 ± 1.74		70.45 ± 1.19		15.68 ± 1.76	
	rs5906883	*A*	72.26 ± 1.01	0.11	75.34 ± 1.27	0.11	71.48 ± 0.84	0.17	14.42 ± 1.06	0.17
		*C*	70.15 ± 1.42		72.70 ± 1.63		70.11 ± 1.16		16.32 ± 1.84	
	rs1465107	*G*	71.14 ± 1.47	0.37	72.74 ± 1.71	0.12	70.51 ± 1.21	0.31	16.11 ± 1.80	0.21
		*A*	71.71 ± 1.00		75.22 ± 1.23		71.24 ± 0.83		14.53 ± 1.08	
	rs1465108	*A*	71.71 ± 1.00	0.37	75.22 ± 1.23	0.12	71.24 ± 0.83	0.31	14.53 ± 1.08	0.21
		*G*	71.14 ± 1.47		72.74 ± 1.71		70.51 ± 1.21		16.11 ± 1.80	
	rs5905809	*C*	70.17 ± 1.37	0.12	72.24 ± 1.60	0.06	69.83 ± 1.13	0.10	16.21 ± 1.80	0.19
		*G*	72.24 ± 1.03		75.58 ± 1.27		71.63 ± 0.85		14.47 ± 1.08	
	rs5906957	*A*	72.24 ± 1.03	0.12	75.58 ± 1.27	0.06	71.63 ± 0.85	0.10	14.47 ± 1.08	0.19
		*G*	70.17 ± 1.37		72.24 ± 1.60		69.83 ± 1.13		16.21 ± 1.80	
	rs6323	*T*	69.48 ± 1.62	0.08	70.88 ± 2.12	**0.02**	69.00 ± 1.34	**0.04**	15.35 ± 2.11	0.43
		*G*	72.20 ± 0.95		75.60 ± 1.12		71.67 ± 0.78		14.95 ± 1.02	
	rs1137070	*C*	70.35 ± 1.46	0.15	73.00 ± 1.66	0.15	70.22 ± 1.18	0.20	17.32 ± 1.77	**0.04**
		*T*	72.15 ± 1.00		75.17 ± 1.26		71.42 ± 0.83		13.91 ± 1.07	
*MAOB*	rs4824562	*A*	71.29 ± 0.95	0.29	73.79 ± 1.10	0.11	70.59 ± 0.77	0.12	14.88 ± 1.04	0.35
		*G*	72.44 ± 1.65		76.85 ± 2.41		72.59 ± 1.43		15.81 ± 2.20	
	rs56220155	*G*	72.08 ± 1.36	0.34	76.86 ± 1.81	0.07	72.67 ± 1.15	0.07	13.82 ± 1.40	0.21
		*A*	71.31 ± 1.02		73.50 ± 1.19		70.38 ± 0.82		15.51 ± 1.17	
	rs2283728	*T*	72.25 ± 1.42	0.32	77.79 ± 1.63	**0.04**	73.04 ± 1.25	0.06	13.73 ± 1.57	0.25
		*C*	71.33 ± 0.98		73.51 ± 1.18		70.45 ± 0.79		15.35 ± 1.09	
	rs2283727	*C*	71.33 ± 0.98	0.32	73.51 ± 1.18	**0.04**	70.45 ± 0.79	0.06	15.35 ± 1.09	0.25
		*A*	72.25 ± 1.42		77.79 ± 1.63		73.04 ± 1.25		13.73 ± 1.57	
	rs3027441	*C*	74.31 ± 1.60	**0.04**	76.83 ± 1.58	0.10	73.72 ± 1.27	**0.02**	14.15 ± 1.74	0.34
		*T*	70.74 ± 0.95		73.74 ± 1.20		70.23 ± 0.78		15.23 ± 1.07	
	rs6324	*C*	70.74 ± 0.95	**0.04**	73.74 ± 1.20	0.10	70.23 ± 0.78	**0.02**	15.23 ± 1.07	0.34
		*T*	74.31 ± 1.60		76.83 ± 1.58		73.72 ± 1.27		14.15 ± 1.74	
	rs3027440	*T*	71.08 ± 0.90	0.12	73.77 ± 1.14	0.08	70.57 ± 0.76	0.08	14.90 ± 1.06	0.34
		*C*	73.73 ± 2.00		77.64 ± 1.83		73.14 ± 1.45		16.00 ± 1.68	

Statistically significant differences are presented in bold

Stratified analysis revealed higher occurrence of *MAOA* 30 bp-uVNTR 3-repeat (*3R*), rs6323 ‘*G*’, and rs1137070 ‘*T*’ variants in the male ADHD probands (Table 4) born to younger mothers (maternal age at pregnancy ≤26 years). Significant risk of association of these variants was also evident from higher relative risk (Table 4). *MAOB* variants failed to show any statistically significant difference (Table 4).

**Table 4:** Analysis of association between maternal age at pregnancy and *MAO* variants of male ADHD probands

Genes	Variants	Alleles	≤ 26 yrs	> 26 yrs	Chi-square (*p*-value)	Relative risk (95% confidence interval)
*MAOA*	30 bp-uVNTR	*3R*	0.76	0.61	4.03 (**0.05**)	1.26 (1.01 – 1.57)
		*4R*	0.24	0.39		
	rs5906883	*A*	0.73	0.61	2.66 (0.10)	–
		*C*	0.27	0.39		
	rs1465107	*G*	0.25	0.36	2.3 (0.13)	–
		*A*	0.75	0.64		
	rs1465108	*A*	0.75	0.64	2.3 (0.13)	–
		*G*	0.25	0.36		
	rs5905809	*C*	0.28	0.39	2.09 (0.15)	–
		*G*	0.72	0.61		
	rs5906957	*A*	0.72	0.61	2.09 (0.15)	–
		*G*	0.28	0.39		
	rs6323	*T*	0.15	0.33	7.22 (**0.007**)	1.27 (1.06 – 1.52)
		*G*	0.85	0.67		
	rs1137070	*C*	0.25	0.40	3.87 (**0.05**)	1.23 (1.00 – 1.54)
		*T*	0.75	0.60		
*MAOB*	rs4824562	*A*	0.83	0.81	0.06 (0.81)	–
		*G*	0.17	0.19		
	rs56220155	*G*	0.33	0.20	3.66 (0.06)	1.19 (1.00 – 1.44)
		*A*	0.67	0.80		
	rs2283728	*T*	0.23	0.16	0.98 (0.32)	–
		*C*	0.77	0.84		
	rs2283727	*C*	0.77	0.84	0.98 (0.32)	–
		*A*	0.23	0.16		
	rs3027441	*C*	0.25	0.15	2.51 (0.11)	–
		*T*	0.75	0.85		
	rs6324	*C*	0.75	0.85	2.51 (0.11)	–
		*T*	0.25	0.15		
	rs3027440	*T*	0.83	0.86	0.31 (0.58)	–
		*C*	0.17	0.14		

Statistically significant differences are presented in bold

## Discussion

Studies on human emphasized a role of MAOA in behavioral attributes, as it is the prime enzyme degrading serotonin, a known regulator of human behavior [63]. Nevertheless, MAOA and MAOB equally degrades dopamine, another monoamine neurotransmitter which also regulates human behavior [64, 65] and interplays with the serotonergic system [66, 67], making both the enzymes crucial while studying human behavior.

Several investigators have tried to find out whether *MAOA* and *MAOB* confer risk of ADHD using family-based association studies, though the data obtained were inconclusive [31, 44–47, 68]. In the Israeli population, family based association studies revealed significant association of *MAOA* 30 bp-uVNTR with ADHD [47]. In the Irish ADHD probands, significantly preferential transmission of *MAOA* rs6323 ‘*G*’ allele and a haplotype was reported, while *MAOB* variants failed to exhibit any association [44]. In European Caucasoid subjects from eight different countries, family based study revealed positive association of *MAOA* with ADHD, while *MAOB* variants failed to do so [45]. In the Taiwanese ADHD population also, significant over-transmission of *MAOA* rs6323 ‘*G*’ allele and higher transmission of the ‘*3R-G*’ (30 bp-uVNTR-rs6323) haplotype was observed [35]. In Caucasian female ADHD probands from USA, a stronger association of *MAOA* variant was reported by family based study [69]. In the Han Chinese population also, preferential transmission of specific alleles and haplotypes to the probands was reported [46]. In the same population, *MAOA* polymorphisms were reported to be transmitted to only the male probands having hyperactive/impulsive subtype [70].

On the contrary, in Caucasian ADHD subjects from the United Kingdom, family based association studies found no significant association of *MAOA* variants with the disorder [31, 71]. A large-scale family based study, recruiting ADHD subjects residing in Ireland and Australia, also failed to identify any significant association of *MAOA* variants [68]. This reported discrepancy in association of *MAOA* and *MAOB* with ADHD could be due to ethnic variations in the frequency of risk variants in the population.

Our earlier investigation on limited number of Indo-Caucasoid ADHD probands (*N* = 73) revealed preferential maternal transmission of the 30 bp-uVNTR ‘*3R*’ allele to the male probands [34]. A follow up study (*N* = 126) revealed biased transmission of ‘*3R-T*’ haplotype (30 bp-uVNTR-rs6323) with high relative risk (8.06e + 007) indicating significant risk of association with ADHD [72]. A later investigation also revealed maternal transmission bias for the ‘*3R-T*’ haplotype to the male probands [73]. In the present family-based study, analysis was conducted on 58 variants located in the *MAOA* and *MAOB* genes. Out of the 58 variants, only 15 were polymorphic in this population. Statistical analysis was conducted on ADHD subjects (*N* = 190) stratified based on gender since *MAO* genes are located on the X-chromosome and dosage is different in the male probands as compared to the females. Significantly preferential maternal transmissions were observed for *MAOA* (rs5905809, rs5906957, rs6323) and *MAOB* (rs56220155, rs2283728, rs2283727, rs3027441, rs6324, rs3027440) variants in the male probands. Maternal transmissions to the male probands were also biased for several haplotypes. The 30 bp-uVNTR ‘*3R*’ allele, as part of haplotypes with other variants, showed transmission bias in the male probands. This observation provided further support to our earlier notion that the ‘30 bp-uVNTR *3R* allele’ could be a risk factor for ADHD and is maternally transmitted to the male probands. This bias may, at least partly, be responsible for the male preponderance of the disorder. Further, preferential transmission of *MAOA* and *MAOB* variants from the mother to the male probands could be responsible for the higher heritability of the disorder, at least in this population. In the female ADHD probands, no such bias in parental transmissions was observed. However, the number of female probands was limited in the present investigation. Further investigation, involving large cohort of subjects from different ethnicity, would help us to elucidate the actual role of MAO in the etiology of ADHD.

In Swedish ADHD probands, the *MAOA* 30 bp-uVNTR ‘*3R*’ allele showed association with disruptive behavior in boys [74]. Our earlier population based analysis on this group of subjects revealed significant contribution of *MAOA* rs6323 [48] and *MAOB* rs56220155 [49] in ADHD associated conduct disorder as well as ODD. The present investigation revealed significant association of *MAOA* rs6323 and rs1137070 as well as *MAOB* rs2283728, rs2283727, rs3027441 and rs6324 with behavioral traits of male ADHD probands. It may be inferred on the basis of the information obtained that variants from both *MAO* genes may contribute to the behavioral traits of ADHD probands warranting further in depth investigation.

This first ever investigation on association between maternal age and *MAO* gene variants revealed statistically significant association between maternal age at pregnancy (≤ 26 years) and three *MAOA* variants, 30 bp-uVNTR ‘*3R*’, rs6323 ‘*G*’, and rs1137070 ‘*T*’ in the male ADHD probands. Out of these three variants, rs6323 also exhibited transmission bias, association with behavioral problems, formed part of haplotypes and was in strong LD with various variants. On the basis of this observation, it may be concluded that these *MAOA* variants, with higher occurrence in probands born to younger mothers, may be contributing to the pathophysiology of ADHD.

### Limitations of the study

The major limitation of the present study was the sample size and hence, further in depth analysis on a large cohort of samples belonging to different ethnic groups would help in validation of the present observation.

## Conclusions

It may be inferred from the data obtained that both *MAOA* and *MAOB* gene variants could be considered as risk factors for ADHD in the Indo-Caucasoid population from eastern India. Our study also revealed association of gene variants with behavioral problems often detected in ADHD subjects and thus could be useful for therapeutic intervention of these subjects. Probands with 30 bp-uVNTR ‘*3R*’ allele may not show improvement in behavioral attributes after treatment with MAOA-inhibitor, since they already possess a compensated amount of the enzyme [75].

## Additional files

Additional file 1: The Hardy-Weinberg equilibrium test performed in female subjects. Description: The table summarizes the genotypic distributions and Hardy-Weinberg equilibrium test performed for *MAO* polymorphic variants in female subjects. (PDF 29 kb)

Additional file 2: Parental allelic transmission in female ADHD probands. Description: The table summarizes the parental allelic transmission of *MAO* alleles in female ADHD probands. (PDF 19 kb)

Additional file 3: Maternal haplotypic transmission to male ADHD probands (only significant data presented). Description: The table summarizes the maternal haplotypic transmission of *MAO* haplotypes to male ADHD probands. (PDF 37 kb)

Additional file 4: Maternal haplotypes not-transmitted to male ADHD probands (only significant data presented). Description: The table summarizes the maternal *MAO* haplotypes not-transmitted to male ADHD probands. (PDF 35 kb)

Additional file 5: Parental haplotypic transmission to female ADHD probands. Description: The table summarizes the parental haplotypic transmission of *MAO* haplotypes to female ADHD probands. (PDF 101 kb)

Additional file 6: Pair-wise Linkage Disequilibrium (LD) pattern of *MAO* polymorphic variants (analyzed using Haploview 4.2). Description: The table summarizes the *D’* and *r* ^*2*^ values for pair-wise LD analysis in male probands, mothers of male probands, female probands and parents of female probands. (PDF 67 kb)

Additional file 7: Analysis of allelic association with CPRS-R ‘T scores’ for oppositional behavior and PACS scores for conduct problems in male ADHD probands. Description: The table summarizes statistical comparisons between the mean scores and *MAO* alleles. (PDF 23 kb)

Additional file 8: Analysis of allelic association with the status of age-of-onset of ADHD in the male probands. Description: The table summarizes statistical comparison of the early and/or late onset of disorder with *MAO* alleles. (PDF 25 kb)

## Abbreviations

ADHD: Attention deficit hyperactivity disorder
bp: Base pair
CPRS-R: Revised conners’ parents rating scale
DNA: Deoxyribonucleic acid
DSM-IV: Diagnostic and statistical manual of mental disorders-4th edition
HHRR: Haplotype-based haplotype relative risk
IQ: Intelligent quotient
LD: Linkage disequilibrium
MAO: Monoamine oxidase
MAOA: Monoamine oxidase A
MAOB: Monoamine oxidase B
ODD: Oppositional defiant disorder
PACS: Parental account of children’s symptoms
PCR: Polymerase chain reaction
PEA: Phenylethylamine
RR: Relative risk or risk ratio
rs: Reference SNP
SNP: Single-nucleotide polymorphism
uVNTR: Upstream variable number of tandem repeats
