# Supplementary material for: Pilot study indicate role of preferentially transmitted monoamine oxidase gene variants in behavioral problems of male ADHD probands
Source: BMC Med Genet. 2017 Oct 5;18:109. doi: 10.1186/s12881-017-0469-5 (PMC5629801; doi:10.1186/s12881-017-0469-5)
Supplement: Supplementary file 1 — The Hardy-Weinberg equilibrium test performed in female subjects. Description: The table summarizes the genotypic distributions and Hardy-Weinberg equilibrium test performed for MAO polymorphic variants in female subjects. (PDF 29 kb) [file 12881_2017_469_MOESM1_ESM.pdf]

Additional file 1: The Hardy-Weinberg equilibrium test performed in female subjects

Genes	Variants	Genotypes	Proband		Mother	
			Count (Frequency)	χ^2 (*p*-value)	Count (Frequency)	χ^2 (*p*-value)
MAOA	30bp-uVNTR	*3R/3R*	9 (0.38)	0.49 (0.48)	78 (0.45)	0.28 (0.60)
		3R/4R	10 (0.42)		74 (0.43)	
		4R/4R	5 (0.20)		21 (0.12)	
	rs5906883	*AA*	10 (0.42)	1.12 (0.29)	73 (0.42)	0.03 (0.86)
		AC	9 (0.38)		78 (0.45)	
		CC	5 (0.20)		22 (0.13)	
	rs1465107	*GG*	5 (0.20)	0.49 (0.48)	21 (0.12)	0.16 (0.69)
		GA	10 (0.42)		82 (0.47)	
		AA	9 (0.38)		70 (0.41)	
	rs1465108	*AA*	9 (0.38)	0.49 (0.48)	70 (0.41)	0.16 (0.69)
		AG	10 (0.42)		82 (0.47)	
		GG	5 (0.20)		21 (0.12)	
	rs5905809	*CC*	9 (0.38)	2.59 (0.11)	23 (0.13)	1.14 (0.29)
		CG	8 (0.33)		89 (0.52)	
		GG	7 (0.29)		61 (0.35)	
	rs5906957	*AA*	7 (0.29)	2.59 (0.11)	61 (0.35)	1.14 (0.29)
		AG	8 (0.33)		89 (0.52)	
		GG	9 (0.38)		23 (0.13)	
	rs6323	*TT*	6 (0.25)	2.37 (0.12)	16 (0.09)	2.26 (0.13)
		TG	8 (0.33)		87 (0.50)	
		GG	10 (0.42)		70 (0.41)	
	rs1137070	*CC*	5 (0.21)	0.11 (0.74)	23 (0.13)	0.01 (0.92)
		CT	11 (0.46)		81 (0.47)	
		TT	8 (0.33)		69 (0.40)	
MAOB	rs4824562	*AA*	14 (0.58)	0.09 (0.76)	102 (0.59)	0.19 (0.66)
		AG	9 (0.38)		63 (0.36)	
		GG	1 (0.04)		8 (0.05)	
	rs56220155	*GG*	1 (0.04)	3.22 (0.07)	21 (0.12)	0.00 (1.00)
		GA	15 (0.63)		79 (0.46)	
		AA	8 (0.33)		73 (0.42)	
	rs2283728	*TT*	1 (0.04)	0.09 (0.76)	15 (0.09)	0.41 (0.52)
		TC	9 (0.38)		66 (0.38)	
		CC	14 (0.58)		92 (0.53)	
	rs2283727	*CC*	14 (0.58)	0.09 (0.76)	92 (0.53)	0.41 (0.52)
		CA	9 (0.38)		66 (0.38)	
		AA	1 (0.04)		15 (0.09)	
	rs3027441	*CC*	1 (0.04)	0.04 (0.84)	13 (0.07)	0.04 (0.84)
		CT	7 (0.29)		67 (0.39)	
		TT	16 (0.67)		93 (0.54)	
	rs6324	*CC*	16 (0.67)	0.04 (0.84)	93 (0.54)	0.04 (0.84)
		CT	7 (0.29)		67 (0.39)	
		TT	1 (0.04)		13 (0.07)	
	rs3027440	*TT*	16 (0.67)	0.04 (0.84)	102 (0.59)	0.01 (0.92)
		TC	7 (0.29)		62 (0.36)	
		CC	1 (0.04)		9 (0.05)	
