# Supplementary material for: Pilot study indicate role of preferentially transmitted monoamine oxidase gene variants in behavioral problems of male ADHD probands
Source: BMC Med Genet. 2017 Oct 5;18:109. doi: 10.1186/s12881-017-0469-5 (PMC5629801; doi:10.1186/s12881-017-0469-5)
Supplement: Supplementary file 2 — Parental allelic transmission in female ADHD probands. Description: The table summarizes the parental allelic transmission of MAO alleles in female ADHD probands. (PDF 19 kb) [file 12881_2017_469_MOESM2_ESM.pdf]

Additional file 2: Parental allelic transmission in female ADHD probands

Genes	Variants	Alleles	Transmitted	Non-transmitted	χ^2 (*p*-value)
MAOA	30bp-uVNTR	*3R*	0.57	0.73	1.22 (0.27)
		4R	0.43	0.27	
	rs5906883	*A*	0.60	0.60	0.00 (1.00)
		C	0.40	0.40	
	rs1465107	*G*	0.40	0.40	0.00 (1.00)
		A	0.60	0.60	
	rs1465108	*A*	0.60	0.60	0.00 (1.00)
		G	0.40	0.40	
	rs5905809	*C*	0.47	0.33	0.74 (0.39)
		G	0.53	0.67	
	rs5906957	*A*	0.53	0.67	0.74 (0.39)
		G	0.47	0.33	
	rs6323	*T*	0.37	0.33	0.05 (0.83)
		G	0.63	0.67	
	rs1137070	*C*	0.40	0.33	0.19 (0.66)
		T	0.60	0.67	
MAOB	rs4824562	*A*	0.77	0.87	0.66 (0.42)
		G	0.23	0.13	
	rs56220155	*G*	0.40	0.20	1.89 (0.17)
		A	0.60	0.80	
	rs2283728	*T*	0.30	0.13	1.62 (0.20)
		C	0.70	0.87	
	rs2283727	*C*	0.70	0.87	1.62 (0.20)
		A	0.30	0.13	
	rs3027441	*C*	0.23	0.20	0.07 (0.80)
		T	0.77	0.80	
	rs6324	*C*	0.77	0.80	0.07 (0.80)
		T	0.23	0.20	
	rs3027440	*T*	0.80	0.80	0.00 (1.00)
		C	0.20	0.20	
