# Supplementary material for: Pilot study indicate role of preferentially transmitted monoamine oxidase gene variants in behavioral problems of male ADHD probands
Source: BMC Med Genet. 2017 Oct 5;18:109. doi: 10.1186/s12881-017-0469-5 (PMC5629801; doi:10.1186/s12881-017-0469-5)
Supplement: Supplementary file 3 — Maternal haplotypic transmission to male ADHD probands (only significant data presented). Description: The table summarizes the maternal haplotypic transmission of MAO haplotypes to male ADHD probands. (PDF 37 kb) [file 12881_2017_469_MOESM3_ESM.pdf]

Additional file 3: Maternal haplotypic transmission to male ADHD probands (only significant data presented)

Variant Combinations	Haplotypes	Transmitted	Non-transmitted	χ^2 (*p*-value)
30bp-uVNTR-rs5905809	*3R-G*	0.63	0.49	5.90 (**0.02**)
30bp-uVNTR-rs5906957	*3R-A*	0.63	0.49	5.90 (**0.02**)
30bp-uVNTR-rs6323	*3R-G*	0.63	0.49	5.34 (**0.02**)
30bp-uVNTR-rs3027440	*3R-T*	0.56	0.44	4.26 (**0.04**)
30bp-uVNTR-rs6324	*3R-C*	0.52	0.41	3.82 (**0.05**)
30bp-uVNTR-rs3027441	*3R-T*	0.52	0.41	3.82 (**0.05**)
30bp-uVNTR-rs2283727	*3R-C*	0.53	0.39	6.41 (**0.01**)
30bp-uVNTR-rs2283728	*3R-C*	0.53	0.39	6.41 (**0.01**)
30bp-uVNTR-rs56220155	*3R-A*	0.48	0.36	4.88 (**0.03**)
rs5906883-rs5905809	*A-G*	0.65	0.52	5.42 (**0.02**)
rs5906883-rs5906957	*A-A*	0.65	0.52	5.42 (**0.02**)
rs5906883-rs6323	*C-G*	0.15	0.05	9.47 (**0.002**)
rs5906883-rs3027440	*A-T*	0.55	0.43	4.27 (**0.04**)
rs5906883-rs2283727	*A-C*	0.51	0.38	5.34 (**0.02**)
rs5906883-rs2283728	*A-C*	0.51	0.38	5.34 (**0.02**)
rs1465107-rs5905809	*A-G*	0.66	0.49	9.09 (**0.003**)
rs1465107-rs5906957	*A-A*	0.66	0.49	9.09 (**0.003**)
rs1465107-rs6323	*A-G*	0.63	0.48	6.44 (**0.01**)
rs1465107-rs3027440	*A-T*	0.57	0.39	8.91 (**0.003**)
rs1465107-rs6324	*A-C*	0.52	0.39	4.79 (**0.03**)
rs1465107-rs3027441	*A-T*	0.52	0.39	4.79 (**0.03**)
rs1465107-rs2283727	*A-C*	0.53	0.37	7.66 (**0.006**)
rs1465107-rs2283728	*A-C*	0.53	0.37	7.66 (**0.006**)
rs1465107-rs56220155	*A-A*	0.47	0.34	5.45 (**0.02**)
rs1465108-rs5905809	*A-G*	0.66	0.49	9.09 (**0.003**)
rs1465108-rs5906957	*A-A*	0.66	0.49	9.09 (**0.003**)
rs1465108-rs6323	*A-G*	0.63	0.48	6.44 (**0.01**)
rs1465108-rs3027440	*A-T*	0.57	0.39	8.91 (**0.003**)
rs1465108-rs6324	*A-C*	0.52	0.39	4.79 (**0.03**)
rs1465108-rs3027441	*A-T*	0.52	0.39	4.79 (**0.03**)
rs1465108-rs2283727	*A-C*	0.53	0.37	7.66 (**0.006**)
rs1465108-rs2283728	*A-C*	0.53	0.37	7.66 (**0.006**)
rs1465108-rs56220155	*A-A*	0.47	0.34	5.45 (**0.02**)
rs5905809-rs5906957	*G-A*	0.67	0.56	4.02 (**0.05**)
rs5905809-rs6323	*G-G*	0.62	0.44	9.63 (**0.002**)
rs5905809-rs1137070	*G-T*	0.63	0.48	7.05 (**0.008**)
rs5905809-rs3027440	*G-T*	0.55	0.36	11.17 (**0.0008**)
rs5905809-rs6324	*G-C*	0.50	0.35	7.12 (**0.008**)
rs5905809-rs3027441	*G-T*	0.50	0.35	7.12 (**0.008**)
rs5905809-rs2283727	*G-C*	0.51	0.32	11.43 (**0.0007**)
rs5905809-rs2283728	*G-C*	0.51	0.32	11.43 (**0.0007**)
rs5905809-rs56220155	*G-A*	0.45	0.30	7.40 (**0.007**)
rs5905809-rs4824562	*G-A*	0.54	0.38	7.63 (**0.006**)

Continued on the next page

Additional file 3: Continued

**Variant Combinations**	**Haplotypes**	**Transmitted**	**Non-transmitted**	**χ^2 (*p*-value)**
rs5906957-rs6323	*A-G*	0.62	0.44	9.63 (**0.002**)
rs5906957-rs1137070	*A-T*	0.63	0.48	7.05 (**0.008**)
rs5906957-rs3027440	*A-T*	0.55	0.36	11.17 (**0.0008**)
rs5906957-rs6324	*A-C*	0.50	0.35	7.12 (**0.008**)
rs5906957-rs3027441	*A-T*	0.50	0.35	7.12 (**0.008**)
rs5906957-rs2283727	*A-C*	0.51	0.32	11.43 (**0.0007**)
rs5906957-rs2283728	*A-C*	0.51	0.32	11.43 (**0.0007**)
rs5906957-rs56220155	*A-A*	0.45	0.30	7.40 (**0.007**)
rs5906957-rs4824562	*A-A*	0.54	0.38	7.63 (**0.006**)
rs6323-rs1137070	*G-T*	0.62	0.48	5.86 (**0.02**)
rs6323-rs3027440	*G-T*	0.62	0.39	16.12 (**5.95E-05**)
rs6323-rs6324	*G-C*	0.57	0.38	11.10 (**0.0009**)
rs6323-rs3027441	*G-T*	0.57	0.38	11.10 (**0.0009**)
rs6323-rs2283727	*G-C*	0.58	0.34	18.15 (**2.05E-05**)
rs6323-rs2283728	*G-C*	0.58	0.34	18.15 (**2.05E-05**)
rs6323-rs56220155	*G-A*	0.51	0.32	12.20 (**0.0005**)
rs1137070-rs3027440	*T-T*	0.55	0.39	7.60 (**0.006**)
rs1137070-rs6324	*T-C*	0.51	0.39	4.31 (**0.04**)
rs1137070-rs3027441	*T-T*	0.51	0.39	4.31 (**0.04**)
rs1137070-rs2283727	*T-C*	0.51	0.35	8.38 (**0.004**)
rs1137070-rs2283728	*T-C*	0.51	0.35	8.38 (**0.004**)
rs1137070-rs56220155	*T-A*	0.46	0.33	5.51 (**0.02**)
rs1137070-rs4824562	*T-A*	0.55	0.44	3.80 (**0.05**)
rs3027440-rs6324	*T-C*	0.80	0.60	14.03 (**0.0002**)
rs3027440-rs3027441	*T-T*	0.80	0.60	14.03 (**0.0002**)
rs3027440-rs2283727	*T-C*	0.80	0.57	18.55 (**1.66E-05**)
rs3027440-rs2283728	*T-C*	0.80	0.57	18.55 (**1.66E-05**)
rs3027440-rs56220155	*T-A*	0.72	0.50	16.01 (**6.30E-05**)
rs3027440-rs4824562	*T-A*	0.66	0.47	11.28 (**0.0008**)
rs6324-rs3027441	*C-T*	0.80	0.65	8.77 (**0.003**)
rs6324-rs2283727	*C-C*	0.80	0.57	17.60 (**2.73E-05**)
rs6324-rs2283728	*C-C*	0.80	0.57	17.60 (**2.73E-05**)
rs6324-rs56220155	*C-A*	0.72	0.52	13.44 (**0.0002**)
rs6324-rs4824562	*C-A*	0.61	0.45	6.99 (**0.008**)
rs3027441-rs2283727	*T-C*	0.80	0.57	17.60 (**2.73E-05**)
rs3027441-rs2283728	*T-C*	0.80	0.57	17.60 (**2.73E-05**)
rs3027441-rs56220155	*T-A*	0.72	0.52	13.44 (**0.0002**)
rs3027441-rs4824562	*T-A*	0.61	0.45	6.99 (**0.008**)
rs2283727-rs2283728	*C-C*	0.81	0.64	11.12 (**0.0009**)
rs2283727-rs56220155	*C-A*	0.74	0.57	9.79 (**0.002**)
rs2283727-rs4824562	*C-A*	0.61	0.44	8.92 (**0.003**)
rs2283728-rs56220155	*C-A*	0.74	0.57	9.79 (**0.002**)
rs2283728-rs4824562	*C-A*	0.61	0.44	8.92 (**0.003**)
rs56220155-rs4824562	*A-A*	0.54	0.38	8.28 (**0.004**)
