# Supplementary material for: Pilot study indicate role of preferentially transmitted monoamine oxidase gene variants in behavioral problems of male ADHD probands
Source: BMC Med Genet. 2017 Oct 5;18:109. doi: 10.1186/s12881-017-0469-5 (PMC5629801; doi:10.1186/s12881-017-0469-5)
Supplement: Supplementary file 4 — Maternal haplotypes not-transmitted to male ADHD probands (only significant data presented). Description: The table summarizes the maternal MAO haplotypes not-transmitted to male ADHD probands. (PDF 35 kb) [file 12881_2017_469_MOESM4_ESM.pdf]

Additional file 4: Maternal haplotypes not-transmitted to male ADHD probands (only significant data presented)

Variant Combinations	Haplotypes	Transmitted	Non-transmitted	χ^2 (*p*-value)
30bp-uVNTR-rs5905809	*3R-C*	0.05	0.16	8.94 (**0.003**)
30bp-uVNTR-rs5906957	*3R-G*	0.05	0.16	8.94 (**0.003**)
30bp-uVNTR-rs6323	*3R-T*	0.06	0.16	7.65 (**0.006**)
30bp-uVNTR-rs3027440	*4R-C*	0.02	0.11	10.49 (**0.001**)
30bp-uVNTR-rs6324	*4R-T*	0.03	0.11	6.19 (**0.01**)
30bp-uVNTR-rs3027441	*4R-C*	0.03	0.11	6.19 (**0.01**)
rs5906883-rs5905809	*A-C*	0.02	0.12	11.51 (**0.0007**)
rs5906883-rs5906957	*A-G*	0.02	0.12	11.51 (**0.0007**)
rs5906883-rs6323	*C-T*	0.18	0.32	7.81 (**0.005**)
rs5906883-rs3027440	*C-C*	0.02	0.11	10.49 (**0.001**)
rs5906883-rs6324	*C-T*	0.03	0.12	9.60 (**0.002**)
rs5906883-rs3027441	*C-C*	0.03	0.12	9.60 (**0.002**)
rs5906883-rs2283727	*C-A*	0.03	0.10	6.79 (**0.009**)
rs5906883-rs2283728	*C-T*	0.03	0.10	6.79 (**0.009**)
rs5906883-rs56220155	*C-G*	0.05	0.14	7.71 (**0.005**)
rs1465107-rs5905809	*A-C*	0.03	0.12	7.95 (**0.005**)
rs1465107-rs5906957	*A-G*	0.03	0.12	7.95 (**0.005**)
rs1465107-rs6323	*G-T*	0.17	0.32	9.36 (**0.002**)
rs1465107-rs3027440	*G-C*	0.02	0.11	10.49 (**0.001**)
rs1465107-rs6324	*G-T*	0.03	0.14	12.60 (**0.0004**)
rs1465107-rs3027441	*G-C*	0.03	0.14	12.60 (**0.0004**)
rs1465107-rs2283727	*G-A*	0.03	0.13	10.58 (**0.001**)
rs1465107-rs2283728	*G-T*	0.03	0.13	10.58 (**0.001**)
rs1465107-rs56220155	*G-G*	0.05	0.16	11.32 (**0.0008**)
rs1465108-rs5905809	*A-C*	0.03	0.12	7.95 (**0.005**)
rs1465108-rs5906957	*A-G*	0.03	0.12	7.95 (**0.005**)
rs1465108-rs6323	*G-T*	0.17	0.32	9.36 (**0.002**)
rs1465108-rs3027440	*G-C*	0.02	0.11	10.49 (**0.001**)
rs1465108-rs6324	*G-T*	0.03	0.14	12.60 (**0.0004**)
rs1465108-rs3027441	*G-C*	0.03	0.14	12.60 (**0.0004**)
rs1465108-rs2283727	*G-A*	0.03	0.13	10.58 (**0.001**)
rs1465108-rs2283728	*G-T*	0.03	0.13	10.58 (**0.001**)
rs1465108-rs56220155	*G-G*	0.05	0.16	11.32 (**0.0008**)
rs5905809-rs6323	*C-T*	0.18	0.33	8.35 (**0.004**)
rs5905809-rs3027440	*C-C*	0.02	0.12	11.51 (**0.0007**)
rs5905809-rs6324	*C-T*	0.03	0.14	12.60 (**0.0004**)
rs5905809-rs3027441	*C-C*	0.03	0.14	12.60 (**0.0004**)
rs5905809-rs2283727	*C-A*	0.03	0.12	9.60 (**0.002**)
rs5905809-rs2283728	*C-T*	0.03	0.12	9.60 (**0.002**)
rs5905809-rs56220155	*C-G*	0.05	0.17	12.27 (**0.0005**)
rs5906957-rs6323	*G-T*	0.18	0.33	8.35 (**0.004**)
rs5906957-rs3027440	*G-C*	0.02	0.12	11.51 (**0.0007**)
rs5906957-rs6324	*G-T*	0.03	0.14	12.60 (**0.0004**)
rs5906957-rs3027441	*G-C*	0.03	0.14	12.60 (**0.0004**)
rs5906957-rs2283727	*G-A*	0.03	0.12	9.60 (**0.002**)
rs5906957-rs2283728	*G-T*	0.03	0.12	9.60 (**0.002**)
rs5906957-rs56220155	*G-G*	0.05	0.17	12.27 (**0.0005**)
rs6323-rs1137070	*T-C*	0.18	0.34	9.93 (**0.002**)
rs6323-rs3027440	*T-C*	0.00	0.16	26.06 (**3.32E-07**)
rs6323-rs6324	*T-T*	0.00	0.17	28.43 (**9.71E-08**)
rs6323-rs3027441	*T-C*	0.00	0.17	28.43 (**9.71E-08**)
rs6323-rs2283727	*T-A*	0.01	0.14	20.74 (**5.25E-06**)
rs6323-rs2283728	*T-T*	0.01	0.14	20.74 (**5.25E-06**)
rs6323-rs56220155	*T-G*	0.01	0.19	26.19 (**3.10E-07**)
rs6323-rs4824562	*T-A*	0.18	0.33	8.35 (**0.004**)

Continued on the next page

Additional file 4: Continued

**Variant Combinations**	**Haplotypes**	**Transmitted**	**Non-transmitted**	χ^2 (***p***-**value**)
rs1137070-rs3027440	*C-C*	0.02	0.13	12.54 (**0.0004**)
rs1137070-rs6324	*C-T*	0.03	0.14	13.63 (**0.0002**)
rs1137070-rs3027441	*C-C*	0.03	0.14	13.63 (**0.0002**)
rs1137070-rs2283727	*C-A*	0.03	0.12	9.60 (**0.002**)
rs1137070-rs2283728	*C-T*	0.03	0.12	9.60 (**0.002**)
rs1137070-rs56220155	*C-G*	0.05	0.16	11.32 (**0.0008**)
rs3027440-rs6324	*C-T*	0.14	0.27	8.11 (**0.004**)
rs3027440-rs3027441	*C-C*	0.14	0.27	8.11 (**0.004**)
rs3027440-rs2283727	*C-A*	0.13	0.25	6.90 (**0.009**)
rs3027440-rs2283728	*C-T*	0.13	0.25	6.90 (**0.009**)
rs3027440-rs56220155	*C-G*	0.13	0.25	6.90 (**0.009**)
rs3027440-rs4824562	*C-A*	0.14	0.26	6.73 (**0.009**)
rs3027440-rs4824562	*C-G*	0.01	0.07	7.64 (**0.006**)
rs6324-rs3027441	*T-C*	0.20	0.35	8.77 (**0.003**)
rs6324-rs2283727	*C-A*	0.01	0.08	9.72 (**0.002**)
rs6324-rs2283728	*C-T*	0.01	0.08	9.72 (**0.002**)
rs6324-rs4824562	*T-G*	0.00	0.07	11.41 (**0.0007**)
rs3027441-rs4824562	*C-G*	0.00	0.07	11.41 (**0.0007**)
rs2283727-rs2283728	*A-T*	0.19	0.36	11.12 (**0.0009**)
rs2283727-rs56220155	*A-G*	0.19	0.36	10.36 (**0.001**)
rs2283727-rs4824562	*A-G*	0.00	0.07	11.41 (**0.0007**)
rs2283728-rs56220155	*T-G*	0.19	0.36	10.36 (**0.001**)
rs2283728-rs4824562	*T-G*	0.00	0.07	11.41 (**0.0007**)
rs56220155-rs4824562	*G-G*	0.00	0.07	11.41 (**0.0007**)
