# Supplementary material for: Pilot study indicate role of preferentially transmitted monoamine oxidase gene variants in behavioral problems of male ADHD probands
Source: BMC Med Genet. 2017 Oct 5;18:109. doi: 10.1186/s12881-017-0469-5 (PMC5629801; doi:10.1186/s12881-017-0469-5)
Supplement: Supplementary file 5 — Parental haplotypic transmission to female ADHD probands. Description: The table summarizes the parental haplotypic transmission of MAO haplotypes to female ADHD probands. (PDF 101 kb) [file 12881_2017_469_MOESM5_ESM.pdf]

Additional file 5: Parental haplotypic transmission to female ADHD probands

Variant Combinations	Haplotypes	Transmitted	Non-transmitted	χ^2 (*p*-value)
30bp-uVNTR-rs5906883	*3R-A*	0.47	0.60	0.71 (0.40)
30bp-uVNTR-rs5906883	*3R-C*	0.10	0.13	0.11 (0.74)
30bp-uVNTR-rs5906883	*4R-A*	0.13	0.00	2.20 (0.14)
30bp-uVNTR-rs5906883	*4R-C*	0.30	0.27	0.05 (0.82)
30bp-uVNTR-rs1465107	*3R-A*	0.47	0.60	0.71 (0.40)
30bp-uVNTR-rs1465107	*3R-G*	0.10	0.13	0.11 (0.74)
30bp-uVNTR-rs1465107	*4R-A*	0.13	0.00	2.20 (0.14)
30bp-uVNTR-rs1465107	*4R-G*	0.30	0.27	0.05 (0.82)
30bp-uVNTR-rs1465108	*3R-A*	0.47	0.60	0.71 (0.40)
30bp-uVNTR-rs1465108	*3R-G*	0.10	0.13	0.11 (0.74)
30bp-uVNTR-rs1465108	*4R-A*	0.13	0.00	2.20 (0.14)
30bp-uVNTR-rs1465108	*4R-G*	0.30	0.27	0.05 (0.82)
30bp-uVNTR-rs5905809	*3R-C*	0.13	0.07	0.45 (0.50)
30bp-uVNTR-rs5905809	*3R-G*	0.43	0.67	2.18 (0.14)
30bp-uVNTR-rs5905809	*4R-C*	0.33	0.27	0.21 (0.65)
30bp-uVNTR-rs5905809	*4R-G*	0.10	0.00	1.61 (0.20)
30bp-uVNTR-rs5906957	*3R-A*	0.43	0.67	2.18 (0.14)
30bp-uVNTR-rs5906957	*3R-G*	0.13	0.07	0.45 (0.50)
30bp-uVNTR-rs5906957	*4R-A*	0.10	0.00	1.61 (0.20)
30bp-uVNTR-rs5906957	*4R-G*	0.33	0.27	0.21 (0.65)
30bp-uVNTR-rs6323	*3R-G*	0.47	0.67	1.61 (0.20)
30bp-uVNTR-rs6323	*3R-T*	0.10	0.07	0.14 (0.71)
30bp-uVNTR-rs6323	*4R-G*	0.17	0.00	2.81 (0.09)
30bp-uVNTR-rs6323	*4R-T*	0.27	0.27	0.00 (1.00)
30bp-uVNTR-rs1137070	*3R-C*	0.13	0.13	0.00 (1.00)
30bp-uVNTR-rs1137070	*3R-T*	0.43	0.60	1.11 (0.29)
30bp-uVNTR-rs1137070	*4R-C*	0.27	0.20	0.24 (0.62)
30bp-uVNTR-rs1137070	*4R-T*	0.17	0.07	0.87 (0.35)
30bp-uVNTR-rs3027440	*3R-C*	0.10	0.20	0.87 (0.35)
30bp-uVNTR-rs3027440	*3R-T*	0.47	0.53	0.18 (0.67)
30bp-uVNTR-rs3027440	*4R-C*	0.10	0.00	1.61 (0.20)
30bp-uVNTR-rs3027440	*4R-T*	0.33	0.27	0.21 (0.65)
30bp-uVNTR-rs6324	*3R-C*	0.43	0.53	0.40 (0.53)
30bp-uVNTR-rs6324	*3R-T*	0.13	0.20	0.34 (0.56)
30bp-uVNTR-rs6324	*4R-C*	0.33	0.27	0.21 (0.65)
30bp-uVNTR-rs6324	*4R-T*	0.10	0.00	1.61 (0.20)
30bp-uVNTR-rs3027441	*3R-C*	0.13	0.20	0.34 (0.56)
30bp-uVNTR-rs3027441	*3R-T*	0.43	0.53	0.40 (0.53)
30bp-uVNTR-rs3027441	*4R-C*	0.10	0.00	1.61 (0.20)
30bp-uVNTR-rs3027441	*4R-T*	0.33	0.27	0.21 (0.65)
30bp-uVNTR-rs2283727	*3R-A*	0.20	0.13	0.30 (0.58)
30bp-uVNTR-rs2283727	*3R-C*	0.37	0.60	2.21 (0.14)
30bp-uVNTR-rs2283727	*4R-A*	0.10	0.00	1.61 (0.20)
30bp-uVNTR-rs2283727	*4R-C*	0.33	0.27	0.21 (0.65)
30bp-uVNTR-rs2283728	*3R-C*	0.37	0.60	2.21 (0.14)
30bp-uVNTR-rs2283728	*3R-T*	0.20	0.13	0.30 (0.58)
30bp-uVNTR-rs2283728	*4R-C*	0.33	0.27	0.21 (0.65)
30bp-uVNTR-rs2283728	*4R-T*	0.10	0.00	1.61 (0.20)
30bp-uVNTR-rs56220155	*3R-A*	0.33	0.60	2.92 (0.09)
30bp-uVNTR-rs56220155	*3R-G*	0.23	0.13	0.63 (0.43)
30bp-uVNTR-rs56220155	*4R-A*	0.27	0.20	0.24 (0.62)
30bp-uVNTR-rs56220155	*4R-G*	0.17	0.07	0.87 (0.35)
30bp-uVNTR-rs4824562	*3R-A*	0.40	0.60	1.61 (0.20)
30bp-uVNTR-rs4824562	*3R-G*	0.17	0.13	0.08 (0.77)
30bp-uVNTR-rs4824562	*4R-A*	0.37	0.27	0.45 (0.50)
30bp-uVNTR-rs4824562	*4R-G*	0.07	0.00	1.05 (0.31)
rs5906883-rs1465107	*A-A*	0.60	0.60	0.00 (1.00)
rs5906883-rs1465107	*C-G*	0.40	0.40	0.00 (1.00)
rs5906883-rs1465108	*A-A*	0.60	0.60	0.00 (1.00)

rs5906883-rs1465108	*C-G*	0.40	0.40	0.00 (1.00)
rs5906883-rs5905809	*A-C*	0.10	0.00	1.61 (0.20)
rs5906883-rs5905809	*A-G*	0.50	0.60	0.40 (0.53)
rs5906883-rs5905809	*C-C*	0.37	0.33	0.05 (0.83)
rs5906883-rs5905809	*C-G*	0.03	0.07	0.26 (0.61)
rs5906883-rs5906957	*A-A*	0.50	0.60	0.40 (0.53)
rs5906883-rs5906957	*A-G*	0.10	0.00	1.61 (0.20)
rs5906883-rs5906957	*C-A*	0.03	0.07	0.26 (0.61)
rs5906883-rs5906957	*C-G*	0.37	0.33	0.05 (0.83)
rs5906883-rs6323	*A-G*	0.53	0.53	0.00 (1.00)
rs5906883-rs6323	*A-T*	0.07	0.07	0.00 (1.00)
rs5906883-rs6323	*C-G*	0.10	0.13	0.11 (0.74)
rs5906883-rs6323	*C-T*	0.30	0.27	0.05 (0.82)
rs5906883-rs1137070	*A-C*	0.10	0.07	0.14 (0.71)
rs5906883-rs1137070	*A-T*	0.50	0.53	0.04 (0.83)
rs5906883-rs1137070	*C-C*	0.30	0.27	0.05 (0.82)
rs5906883-rs1137070	*C-T*	0.10	0.13	0.11 (0.74)
rs5906883-rs3027440	*A-C*	0.13	0.20	0.34 (0.56)
rs5906883-rs3027440	*A-T*	0.47	0.40	0.18 (0.67)
rs5906883-rs3027440	*C-C*	0.07	0.00	1.05 (0.31)
rs5906883-rs3027440	*C-T*	0.33	0.40	0.19 (0.66)
rs5906883-rs6324	*A-C*	0.43	0.40	0.05 (0.83)
rs5906883-rs6324	*A-T*	0.17	0.20	0.08 (0.78)
rs5906883-rs6324	*C-C*	0.33	0.40	0.19 (0.66)
rs5906883-rs6324	*C-T*	0.07	0.00	1.05 (0.31)
rs5906883-rs3027441	*A-C*	0.17	0.20	0.08 (0.78)
rs5906883-rs3027441	*A-T*	0.43	0.40	0.05 (0.83)
rs5906883-rs3027441	*C-C*	0.07	0.00	1.05 (0.31)
rs5906883-rs3027441	*C-T*	0.33	0.40	0.19 (0.66)
rs5906883-rs2283727	*A-A*	0.23	0.13	0.63 (0.43)
rs5906883-rs2283727	*A-C*	0.37	0.47	0.42 (0.52)
rs5906883-rs2283727	*C-A*	0.07	0.00	1.05 (0.31)
rs5906883-rs2283727	*C-C*	0.33	0.40	0.19 (0.66)
rs5906883-rs2283728	*A-C*	0.37	0.47	0.42 (0.52)
rs5906883-rs2283728	*A-T*	0.23	0.13	0.63 (0.43)
rs5906883-rs2283728	*C-C*	0.33	0.40	0.19 (0.66)
rs5906883-rs2283728	*C-T*	0.07	0.00	1.05 (0.31)
rs5906883-rs56220155	*A-A*	0.37	0.47	0.42 (0.52)
rs5906883-rs56220155	*A-G*	0.23	0.13	0.63 (0.43)
rs5906883-rs56220155	*C-A*	0.23	0.33	0.51 (0.47)
rs5906883-rs56220155	*C-G*	0.17	0.07	0.87 (0.35)
rs5906883-rs4824562	*A-A*	0.40	0.53	0.72 (0.40)
rs5906883-rs4824562	*A-G*	0.20	0.07	1.35 (0.24)
rs5906883-rs4824562	*C-A*	0.37	0.33	0.05 (0.83)
rs5906883-rs4824562	*C-G*	0.03	0.07	0.26 (0.61)
rs1465107-rs1465108	*A-A*	0.60	0.60	0.00 (1.00)
rs1465107-rs1465108	*G-G*	0.40	0.40	0.00 (1.00)
rs1465107-rs5905809	*A-C*	0.10	0.00	1.61 (0.20)
rs1465107-rs5905809	*A-G*	0.50	0.60	0.40 (0.53)
rs1465107-rs5905809	*G-C*	0.37	0.33	0.05 (0.83)
rs1465107-rs5905809	*G-G*	0.03	0.07	0.26 (0.61)
rs1465107-rs5906957	*A-A*	0.50	0.60	0.40 (0.53)
rs1465107-rs5906957	*A-G*	0.10	0.00	1.61 (0.20)
rs1465107-rs5906957	*G-A*	0.03	0.07	0.26 (0.61)
rs1465107-rs5906957	*G-G*	0.37	0.33	0.05 (0.83)
rs1465107-rs6323	*A-G*	0.53	0.53	0.00 (1.00)
rs1465107-rs6323	*A-T*	0.07	0.07	0.00 (1.00)
rs1465107-rs6323	*G-G*	0.10	0.13	0.11 (0.74)
rs1465107-rs6323	*G-T*	0.30	0.27	0.05 (0.82)
rs1465107-rs1137070	*A-C*	0.10	0.07	0.14 (0.71)
rs1465107-rs1137070	*A-T*	0.50	0.53	0.04 (0.83)
rs1465107-rs1137070	*G-C*	0.30	0.27	0.05 (0.82)

rs1465107-rs1137070	*G-T*	0.10	0.13	0.11 (0.74)
rs1465107-rs3027440	*A-C*	0.13	0.20	0.34 (0.56)
rs1465107-rs3027440	*A-T*	0.47	0.40	0.18 (0.67)
rs1465107-rs3027440	*G-C*	0.07	0.00	1.05 (0.31)
rs1465107-rs3027440	*G-T*	0.33	0.40	0.19 (0.66)
rs1465107-rs6324	*A-C*	0.43	0.40	0.05 (0.83)
rs1465107-rs6324	*A-T*	0.17	0.20	0.08 (0.78)
rs1465107-rs6324	*G-C*	0.33	0.40	0.19 (0.66)
rs1465107-rs6324	*G-T*	0.07	0.00	1.05 (0.31)
rs1465107-rs3027441	*A-C*	0.17	0.20	0.08 (0.78)
rs1465107-rs3027441	*A-T*	0.43	0.40	0.05 (0.83)
rs1465107-rs3027441	*G-C*	0.07	0.00	1.05 (0.31)
rs1465107-rs3027441	*G-T*	0.33	0.40	0.19 (0.66)
rs1465107-rs2283727	*A-A*	0.23	0.13	0.63 (0.43)
rs1465107-rs2283727	*A-C*	0.37	0.47	0.42 (0.52)
rs1465107-rs2283727	*G-A*	0.07	0.00	1.05 (0.31)
rs1465107-rs2283727	*G-C*	0.33	0.40	0.19 (0.66)
rs1465107-rs2283728	*A-C*	0.37	0.47	0.42 (0.52)
rs1465107-rs2283728	*A-T*	0.23	0.13	0.63 (0.43)
rs1465107-rs2283728	*G-C*	0.33	0.40	0.19 (0.66)
rs1465107-rs2283728	*G-T*	0.07	0.00	1.05 (0.31)
rs1465107-rs56220155	*A-A*	0.37	0.47	0.42 (0.52)
rs1465107-rs56220155	*A-G*	0.23	0.13	0.63 (0.43)
rs1465107-rs56220155	*G-A*	0.23	0.33	0.51 (0.47)
rs1465107-rs56220155	*G-G*	0.17	0.07	0.87 (0.35)
rs1465107-rs4824562	*A-A*	0.40	0.53	0.72 (0.40)
rs1465107-rs4824562	*A-G*	0.20	0.07	1.35 (0.24)
rs1465107-rs4824562	*G-A*	0.37	0.33	0.05 (0.83)
rs1465107-rs4824562	*G-G*	0.03	0.07	0.26 (0.61)
rs1465108-rs5905809	*A-C*	0.10	0.00	1.61 (0.20)
rs1465108-rs5905809	*A-G*	0.50	0.60	0.40 (0.53)
rs1465108-rs5905809	*G-C*	0.37	0.33	0.05 (0.83)
rs1465108-rs5905809	*G-G*	0.03	0.07	0.26 (0.61)
rs1465108-rs5906957	*A-A*	0.50	0.60	0.40 (0.53)
rs1465108-rs5906957	*A-G*	0.10	0.00	1.61 (0.20)
rs1465108-rs5906957	*G-A*	0.03	0.07	0.26 (0.61)
rs1465108-rs5906957	*G-G*	0.37	0.33	0.05 (0.83)
rs1465108-rs6323	*A-G*	0.53	0.53	0.00 (1.00)
rs1465108-rs6323	*A-T*	0.07	0.07	0.00 (1.00)
rs1465108-rs6323	*G-G*	0.10	0.13	0.11 (0.74)
rs1465108-rs6323	*G-T*	0.30	0.27	0.05 (0.82)
rs1465108-rs1137070	*A-C*	0.10	0.07	0.14 (0.71)
rs1465108-rs1137070	*A-T*	0.50	0.53	0.04 (0.83)
rs1465108-rs1137070	*G-C*	0.30	0.27	0.05 (0.82)
rs1465108-rs1137070	*G-T*	0.10	0.13	0.11 (0.74)
rs1465108-rs3027440	*A-C*	0.13	0.20	0.34 (0.56)
rs1465108-rs3027440	*A-T*	0.47	0.40	0.18 (0.67)
rs1465108-rs3027440	*G-C*	0.07	0.00	1.05 (0.31)
rs1465108-rs3027440	*G-T*	0.33	0.40	0.19 (0.66)
rs1465108-rs6324	*A-C*	0.43	0.40	0.05 (0.83)
rs1465108-rs6324	*A-T*	0.17	0.20	0.08 (0.78)
rs1465108-rs6324	*G-C*	0.33	0.40	0.19 (0.66)
rs1465108-rs6324	*G-T*	0.07	0.00	1.05 (0.31)
rs1465108-rs3027441	*A-C*	0.17	0.20	0.08 (0.78)
rs1465108-rs3027441	*A-T*	0.43	0.40	0.05 (0.83)
rs1465108-rs3027441	*G-C*	0.07	0.00	1.05 (0.31)
rs1465108-rs3027441	*G-T*	0.33	0.40	0.19 (0.66)
rs1465108-rs2283727	*A-A*	0.23	0.13	0.63 (0.43)
rs1465108-rs2283727	*A-C*	0.37	0.47	0.42 (0.52)
rs1465108-rs2283727	*G-A*	0.07	0.00	1.05 (0.31)
rs1465108-rs2283727	*G-C*	0.33	0.40	0.19 (0.66)
rs1465108-rs2283728	*A-C*	0.37	0.47	0.42 (0.52)

rs1465108-rs2283728	*A-T*	0.23	0.13	0.63 (0.43)
rs1465108-rs2283728	*G-C*	0.33	0.40	0.19 (0.66)
rs1465108-rs2283728	*G-T*	0.07	0.00	1.05 (0.31)
rs1465108-rs56220155	*A-A*	0.37	0.47	0.42 (0.52)
rs1465108-rs56220155	*A-G*	0.23	0.13	0.63 (0.43)
rs1465108-rs56220155	*G-A*	0.23	0.33	0.51 (0.47)
rs1465108-rs56220155	*G-G*	0.17	0.07	0.87 (0.35)
rs1465108-rs4824562	*A-A*	0.40	0.53	0.72 (0.40)
rs1465108-rs4824562	*A-G*	0.20	0.07	1.35 (0.24)
rs1465108-rs4824562	*G-A*	0.37	0.33	0.05 (0.83)
rs1465108-rs4824562	*G-G*	0.03	0.07	0.26 (0.61)
rs5905809-rs5906957	*C-G*	0.47	0.33	0.73 (0.39)
rs5905809-rs5906957	*G-A*	0.53	0.67	0.73 (0.39)
rs5905809-rs6323	*C-G*	0.10	0.07	0.14 (0.71)
rs5905809-rs6323	*C-T*	0.37	0.27	0.45 (0.50)
rs5905809-rs6323	*G-G*	0.53	0.60	0.18 (0.67)
rs5905809-rs6323	*G-T*	0.00	0.07	2.05 (0.15)
rs5905809-rs1137070	*C-C*	0.30	0.20	0.51 (0.47)
rs5905809-rs1137070	*C-T*	0.17	0.13	0.08 (0.77)
rs5905809-rs1137070	*G-C*	0.10	0.13	0.11 (0.74)
rs5905809-rs1137070	*G-T*	0.43	0.53	0.40 (0.53)
rs5905809-rs3027440	*C-C*	0.07	0.00	1.05 (0.31)
rs5905809-rs3027440	*C-T*	0.40	0.33	0.19 (0.66)
rs5905809-rs3027440	*G-C*	0.13	0.20	0.34 (0.56)
rs5905809-rs3027440	*G-T*	0.40	0.47	0.18 (0.67)
rs5905809-rs6324	*C-C*	0.40	0.33	0.19 (0.66)
rs5905809-rs6324	*C-T*	0.07	0.00	1.05 (0.31)
rs5905809-rs6324	*G-C*	0.37	0.47	0.42 (0.52)
rs5905809-rs6324	*G-T*	0.17	0.20	0.08 (0.78)
rs5905809-rs3027441	*C-C*	0.07	0.00	1.05 (0.31)
rs5905809-rs3027441	*C-T*	0.40	0.33	0.19 (0.66)
rs5905809-rs3027441	*G-C*	0.17	0.20	0.08 (0.78)
rs5905809-rs3027441	*G-T*	0.37	0.47	0.42 (0.52)
rs5905809-rs2283727	*C-A*	0.10	0.00	1.61 (0.20)
rs5905809-rs2283727	*C-C*	0.37	0.33	0.05 (0.83)
rs5905809-rs2283727	*G-A*	0.20	0.13	0.30 (0.58)
rs5905809-rs2283727	*G-C*	0.33	0.53	1.67 (0.20)
rs5905809-rs2283728	*C-C*	0.37	0.33	0.05 (0.83)
rs5905809-rs2283728	*C-T*	0.10	0.00	1.61 (0.20)
rs5905809-rs2283728	*G-C*	0.33	0.53	1.67 (0.20)
rs5905809-rs2283728	*G-T*	0.20	0.13	0.30 (0.58)
rs5905809-rs56220155	*C-A*	0.27	0.27	0.00 (1.00)
rs5905809-rs56220155	*C-G*	0.20	0.07	1.35 (0.24)
rs5905809-rs56220155	*G-A*	0.33	0.53	1.67 (0.20)
rs5905809-rs56220155	*G-G*	0.20	0.13	0.30 (0.58)
rs5905809-rs4824562	*C-A*	0.43	0.27	1.18 (0.28)
rs5905809-rs4824562	*C-G*	0.03	0.07	0.26 (0.61)
rs5905809-rs4824562	*G-A*	0.33	0.60	2.92 (0.09)
rs5905809-rs4824562	*G-G*	0.20	0.07	1.35 (0.24)
rs5906957-rs6323	*A-G*	0.53	0.60	0.18 (0.67)
rs5906957-rs6323	*A-T*	0.00	0.07	2.05 (0.15)
rs5906957-rs6323	*G-G*	0.10	0.07	0.14 (0.71)
rs5906957-rs6323	*G-T*	0.37	0.27	0.45 (0.50)
rs5906957-rs1137070	*A-C*	0.10	0.13	0.11 (0.74)
rs5906957-rs1137070	*A-T*	0.43	0.53	0.40 (0.53)
rs5906957-rs1137070	*G-C*	0.30	0.20	0.51 (0.47)
rs5906957-rs1137070	*G-T*	0.17	0.13	0.08 (0.77)
rs5906957-rs3027440	*A-C*	0.13	0.20	0.34 (0.56)
rs5906957-rs3027440	*A-T*	0.40	0.47	0.18 (0.67)
rs5906957-rs3027440	*G-C*	0.07	0.00	1.05 (0.31)
rs5906957-rs3027440	*G-T*	0.40	0.33	0.19 (0.66)
rs5906957-rs6324	*A-C*	0.37	0.47	0.42 (0.52)

rs5906957-rs6324	*A-T*	0.17	0.20	0.08 (0.78)
rs5906957-rs6324	*G-C*	0.40	0.33	0.19 (0.66)
rs5906957-rs6324	*G-T*	0.07	0.00	1.05 (0.31)
rs5906957-rs3027441	*A-C*	0.17	0.20	0.08 (0.78)
rs5906957-rs3027441	*A-T*	0.37	0.47	0.42 (0.52)
rs5906957-rs3027441	*G-C*	0.07	0.00	1.05 (0.31)
rs5906957-rs3027441	*G-T*	0.40	0.33	0.19 (0.66)
rs5906957-rs2283727	*A-A*	0.20	0.13	0.30 (0.58)
rs5906957-rs2283727	*A-C*	0.33	0.53	1.67 (0.20)
rs5906957-rs2283727	*G-A*	0.10	0.00	1.61 (0.20)
rs5906957-rs2283727	*G-C*	0.37	0.33	0.05 (0.83)
rs5906957-rs2283728	*A-C*	0.33	0.53	1.67 (0.20)
rs5906957-rs2283728	*A-T*	0.20	0.13	0.30 (0.58)
rs5906957-rs2283728	*G-C*	0.37	0.33	0.05 (0.83)
rs5906957-rs2283728	*G-T*	0.10	0.00	1.61 (0.20)
rs5906957-rs56220155	*A-A*	0.33	0.53	1.67 (0.20)
rs5906957-rs56220155	*A-G*	0.20	0.13	0.30 (0.58)
rs5906957-rs56220155	*G-A*	0.27	0.27	0.00 (1.00)
rs5906957-rs56220155	*G-G*	0.20	0.07	1.35 (0.24)
rs5906957-rs4824562	*A-A*	0.33	0.60	2.92 (0.09)
rs5906957-rs4824562	*A-G*	0.20	0.07	1.35 (0.24)
rs5906957-rs4824562	*G-A*	0.43	0.27	1.18 (0.28)
rs5906957-rs4824562	*G-G*	0.03	0.07	0.26 (0.61)
rs6323-rs1137070	*G-C*	0.17	0.13	0.08 (0.77)
rs6323-rs1137070	*G-T*	0.47	0.53	0.18 (0.67)
rs6323-rs1137070	*T-C*	0.23	0.20	0.06 (0.80)
rs6323-rs1137070	*T-T*	0.13	0.13	0.00 (1.00)
rs6323-rs3027440	*G-C*	0.13	0.20	0.34 (0.56)
rs6323-rs3027440	*G-T*	0.50	0.47	0.04 (0.83)
rs6323-rs3027440	*T-C*	0.07	0.00	1.05 (0.31)
rs6323-rs3027440	*T-T*	0.30	0.33	0.05 (0.82)
rs6323-rs6324	*G-C*	0.47	0.47	0.00 (1.00)
rs6323-rs6324	*G-T*	0.17	0.20	0.08 (0.78)
rs6323-rs6324	*T-C*	0.30	0.33	0.05 (0.82)
rs6323-rs6324	*T-T*	0.07	0.00	1.05 (0.31)
rs6323-rs3027441	*G-C*	0.17	0.20	0.08 (0.78)
rs6323-rs3027441	*G-T*	0.47	0.47	0.00 (1.00)
rs6323-rs3027441	*T-C*	0.07	0.00	1.05 (0.31)
rs6323-rs3027441	*T-T*	0.30	0.33	0.05 (0.82)
rs6323-rs2283727	*G-A*	0.27	0.13	1.03 (0.31)
rs6323-rs2283727	*G-C*	0.37	0.53	1.14 (0.29)
rs6323-rs2283727	*T-A*	0.03	0.00	0.51 (0.47)
rs6323-rs2283727	*T-C*	0.33	0.33	0.00 (1.00)
rs6323-rs2283728	*G-C*	0.37	0.53	1.14 (0.29)
rs6323-rs2283728	*G-T*	0.27	0.13	1.03 (0.31)
rs6323-rs2283728	*T-C*	0.33	0.33	0.00 (1.00)
rs6323-rs2283728	*T-T*	0.03	0.00	0.51 (0.47)
rs6323-rs56220155	*G-A*	0.37	0.53	1.14 (0.29)
rs6323-rs56220155	*G-G*	0.27	0.13	1.03 (0.31)
rs6323-rs56220155	*T-A*	0.23	0.27	0.06 (0.81)
rs6323-rs56220155	*T-G*	0.13	0.07	0.45 (0.50)
rs6323-rs4824562	*G-A*	0.43	0.53	0.40 (0.53)
rs6323-rs4824562	*G-G*	0.20	0.13	0.30 (0.58)
rs6323-rs4824562	*T-A*	0.33	0.33	0.00 (1.00)
rs6323-rs4824562	*T-G*	0.03	0.00	0.51 (0.47)
rs1137070-rs3027440	*C-C*	0.03	0.00	0.51 (0.47)
rs1137070-rs3027440	*C-T*	0.37	0.33	0.05 (0.83)
rs1137070-rs3027440	*T-C*	0.17	0.20	0.08 (0.78)
rs1137070-rs3027440	*T-T*	0.43	0.47	0.05 (0.83)
rs1137070-rs6324	*C-C*	0.33	0.33	0.00 (1.00)
rs1137070-rs6324	*C-T*	0.07	0.00	1.05 (0.31)
rs1137070-rs6324	*T-C*	0.43	0.47	0.05 (0.83)

rs1137070-rs6324	*T-T*	0.17	0.20	0.08 (0.78)
rs1137070-rs3027441	*C-C*	0.07	0.00	1.05 (0.31)
rs1137070-rs3027441	*C-T*	0.33	0.33	0.00 (1.00)
rs1137070-rs3027441	*T-C*	0.17	0.20	0.08 (0.78)
rs1137070-rs3027441	*T-T*	0.43	0.47	0.05 (0.83)
rs1137070-rs2283727	*C-A*	0.07	0.00	1.05 (0.31)
rs1137070-rs2283727	*C-C*	0.33	0.33	0.00 (1.00)
rs1137070-rs2283727	*T-A*	0.23	0.13	0.63 (0.43)
rs1137070-rs2283727	*T-C*	0.37	0.53	1.14 (0.29)
rs1137070-rs2283728	*C-C*	0.33	0.33	0.00 (1.00)
rs1137070-rs2283728	*C-T*	0.07	0.00	1.05 (0.31)
rs1137070-rs2283728	*T-C*	0.37	0.53	1.14 (0.29)
rs1137070-rs2283728	*T-T*	0.23	0.13	0.63 (0.43)
rs1137070-rs56220155	*C-A*	0.23	0.27	0.06 (0.81)
rs1137070-rs56220155	*C-G*	0.17	0.07	0.87 (0.35)
rs1137070-rs56220155	*T-A*	0.37	0.53	1.14 (0.29)
rs1137070-rs56220155	*T-G*	0.23	0.13	0.63 (0.43)
rs1137070-rs4824562	*C-A*	0.37	0.33	0.05 (0.83)
rs1137070-rs4824562	*C-G*	0.03	0.00	0.51 (0.47)
rs1137070-rs4824562	*T-A*	0.40	0.53	0.72 (0.40)
rs1137070-rs4824562	*T-G*	0.20	0.13	0.30 (0.58)
rs3027440-rs6324	*C-T*	0.20	0.20	0.00 (1.00)
rs3027440-rs6324	*T-C*	0.77	0.80	0.06 (0.80)
rs3027440-rs6324	*T-T*	0.03	0.00	0.51 (0.47)
rs3027440-rs3027441	*C-C*	0.20	0.20	0.00 (1.00)
rs3027440-rs3027441	*T-C*	0.03	0.00	0.51 (0.47)
rs3027440-rs3027441	*T-T*	0.77	0.80	0.06 (0.80)
rs3027440-rs2283727	*C-A*	0.17	0.13	0.08 (0.77)
rs3027440-rs2283727	*C-C*	0.03	0.07	0.26 (0.61)
rs3027440-rs2283727	*T-A*	0.13	0.00	2.20 (0.14)
rs3027440-rs2283727	*T-C*	0.67	0.80	0.87 (0.35)
rs3027440-rs2283728	*C-C*	0.03	0.07	0.26 (0.61)
rs3027440-rs2283728	*C-T*	0.17	0.13	0.08 (0.77)
rs3027440-rs2283728	*T-C*	0.67	0.80	0.87 (0.35)
rs3027440-rs2283728	*T-T*	0.13	0.00	2.20 (0.14)
rs3027440-rs56220155	*C-A*	0.03	0.07	0.26 (0.61)
rs3027440-rs56220155	*C-G*	0.17	0.13	0.08 (0.77)
rs3027440-rs56220155	*T-A*	0.57	0.73	1.18 (0.28)
rs3027440-rs56220155	*T-G*	0.23	0.07	1.90 (0.17)
rs3027440-rs4824562	*C-A*	0.20	0.20	0.00 (1.00)
rs3027440-rs4824562	*T-A*	0.57	0.67	0.42 (0.52)
rs3027440-rs4824562	*T-G*	0.23	0.13	0.63 (0.43)
rs6324-rs3027441	*C-T*	0.77	0.80	0.06 (0.80)
rs6324-rs3027441	*T-C*	0.23	0.20	0.06 (0.80)
rs6324-rs2283727	*C-A*	0.10	0.00	1.61 (0.20)
rs6324-rs2283727	*C-C*	0.67	0.80	0.87 (0.35)
rs6324-rs2283727	*T-A*	0.20	0.13	0.30 (0.58)
rs6324-rs2283727	*T-C*	0.03	0.07	0.26 (0.61)
rs6324-rs2283728	*C-C*	0.67	0.80	0.87 (0.35)
rs6324-rs2283728	*C-T*	0.10	0.00	1.61 (0.20)
rs6324-rs2283728	*T-C*	0.03	0.07	0.26 (0.61)
rs6324-rs2283728	*T-T*	0.20	0.13	0.30 (0.58)
rs6324-rs56220155	*C-A*	0.57	0.73	1.18 (0.28)
rs6324-rs56220155	*C-G*	0.20	0.07	1.35 (0.24)
rs6324-rs56220155	*T-A*	0.03	0.07	0.26 (0.61)
rs6324-rs56220155	*T-G*	0.20	0.13	0.30 (0.58)
rs6324-rs4824562	*C-A*	0.53	0.67	0.73 (0.39)
rs6324-rs4824562	*C-G*	0.23	0.13	0.63 (0.43)
rs6324-rs4824562	*T-A*	0.23	0.20	0.06 (0.80)
rs3027441-rs2283727	*C-A*	0.20	0.13	0.30 (0.58)
rs3027441-rs2283727	*C-C*	0.03	0.07	0.26 (0.61)
rs3027441-rs2283727	*T-A*	0.10	0.00	1.61 (0.20)

rs3027441-rs2283727	*T-C*	0.67	0.80	0.87 (0.35)
rs3027441-rs2283728	*C-C*	0.03	0.07	0.26 (0.61)
rs3027441-rs2283728	*C-T*	0.20	0.13	0.30 (0.58)
rs3027441-rs2283728	*T-C*	0.67	0.80	0.87 (0.35)
rs3027441-rs2283728	*T-T*	0.10	0.00	1.61 (0.20)
rs3027441-rs56220155	*C-A*	0.03	0.07	0.26 (0.61)
rs3027441-rs56220155	*C-G*	0.20	0.13	0.30 (0.58)
rs3027441-rs56220155	*T-A*	0.57	0.73	1.18 (0.28)
rs3027441-rs56220155	*T-G*	0.20	0.07	1.35 (0.24)
rs3027441-rs4824562	*C-A*	0.23	0.20	0.06 (0.80)
rs3027441-rs4824562	*T-A*	0.53	0.67	0.73 (0.39)
rs3027441-rs4824562	*T-G*	0.23	0.13	0.63 (0.43)
rs2283727-rs2283728	*A-T*	0.30	0.13	1.50 (0.22)
rs2283727-rs2283728	*C-C*	0.70	0.87	1.50 (0.22)
rs2283727-rs56220155	*A-G*	0.30	0.13	1.50 (0.22)
rs2283727-rs56220155	*C-A*	0.60	0.80	1.80 (0.18)
rs2283727-rs56220155	*C-G*	0.10	0.07	0.14 (0.71)
rs2283727-rs4824562	*A-A*	0.27	0.13	1.03 (0.31)
rs2283727-rs4824562	*A-G*	0.03	0.00	0.51 (0.47)
rs2283727-rs4824562	*C-A*	0.50	0.73	2.23 (0.14)
rs2283727-rs4824562	*C-G*	0.20	0.13	0.30 (0.58)
rs2283728-rs56220155	*C-A*	0.60	0.80	1.80 (0.18)
rs2283728-rs56220155	*C-G*	0.10	0.07	0.14 (0.71)
rs2283728-rs56220155	*T-G*	0.30	0.13	1.50 (0.22)
rs2283728-rs4824562	*C-A*	0.50	0.73	2.23 (0.14)
rs2283728-rs4824562	*C-G*	0.20	0.13	0.30 (0.58)
rs2283728-rs4824562	*T-A*	0.27	0.13	1.03 (0.31)
rs2283728-rs4824562	*T-G*	0.03	0.00	0.51 (0.47)
rs56220155-rs4824562	*A-A*	0.40	0.67	2.85 (0.09)
rs56220155-rs4824562	*A-G*	0.20	0.13	0.30 (0.58)
rs56220155-rs4824562	*G-A*	0.37	0.20	1.30 (0.25)
rs56220155-rs4824562	*G-G*	0.03	0.00	0.51 (0.47)
