# Supplementary material for: Pilot study indicate role of preferentially transmitted monoamine oxidase gene variants in behavioral problems of male ADHD probands
Source: BMC Med Genet. 2017 Oct 5;18:109. doi: 10.1186/s12881-017-0469-5 (PMC5629801; doi:10.1186/s12881-017-0469-5)
Supplement: Supplementary file 6 — Pair-wise Linkage Disequilibrium (LD) pattern of MAO polymorphic variants (analyzed using Haploview 4.2). Description: The table summarizes the D’ and r 2 values for pair-wise LD analysis in male probands, mothers of male probands, female probands and parents of female probands. (PDF 67 kb) [file 12881_2017_469_MOESM6_ESM.pdf]

**Additional file 6: Pair-wise Linkage Disequilibrium (LD) pattern of *MAO* polymorphic variants (analyzed using Haploview 4.2)**

| *D′* / $r^2$ | 30bp-uVNTR | rs5906883 | rs1465107 | rs1465108 | rs5905809 | rs5906957 | rs6323 | rs1137070 | rs3027440 | rs6324 | rs3027441 | rs2283727 | rs2283728 | rs56220155 | rs4824562 |
|---|---|---|---|---|---|---|---|---|---|---|---|---|---|---|---|
| **Male proband** | | | | | | | | | | | | | | | |
| **30bp-uVNTR** | - | 0.80 | 0.77 | 0.77 | 0.82 | 0.82 | 0.61 | 0.74 | 0.62 | 0.52 | 0.52 | 0.39 | 0.39 | 0.32 | 0.00 |
| **rs5906883** | 0.59 | - | 0.97 | 0.97 | 0.91 | 0.91 | 0.64 | 0.86 | 0.64 | 0.55 | 0.55 | 0.61 | 0.61 | 0.50 | 0.02 |
| **rs1465107** | 0.60 | 0.87 | - | 1.00 | 0.94 | 0.94 | 0.61 | 0.91 | 0.62 | 0.52 | 0.52 | 0.59 | 0.59 | 0.47 | 0.00 |
| **rs1465108** | 0.60 | 0.87 | 1.00 | - | 0.94 | 0.94 | 0.61 | 0.91 | 0.62 | 0.52 | 0.52 | 0.59 | 0.59 | 0.47 | 0.00 |
| **rs5905809** | 0.59 | 0.79 | 0.77 | 0.77 | - | 1.00 | 0.70 | 0.83 | 0.65 | 0.56 | 0.56 | 0.63 | 0.63 | 0.52 | 0.07 |
| **rs5906957** | 0.59 | 0.79 | 0.77 | 0.77 | 1.00 | - | 0.70 | 0.83 | 0.65 | 0.56 | 0.56 | 0.63 | 0.63 | 0.52 | 0.07 |
| **rs6323** | 0.27 | 0.27 | 0.27 | 0.27 | 0.31 | 0.31 | - | 0.67 | 1.00 | 0.88 | 0.88 | 0.87 | 0.87 | 0.81 | 0.00 |
| **rs1137070** | 0.51 | 0.74 | 0.77 | 0.77 | 0.66 | 0.66 | 0.30 | - | 0.64 | 0.55 | 0.55 | 0.61 | 0.61 | 0.50 | 0.02 |
| **rs3027440** | 0.03 | 0.03 | 0.03 | 0.03 | 0.04 | 0.04 | 0.06 | 0.03 | - | 0.90 | 0.90 | 0.85 | 0.85 | 0.83 | 0.78 |
| **rs6324** | 0.03 | 0.03 | 0.03 | 0.03 | 0.04 | 0.04 | 0.06 | 0.03 | 0.57 | - | 1.00 | 0.91 | 0.91 | 0.83 | 1.00 |
| **rs3027441** | 0.03 | 0.03 | 0.03 | 0.03 | 0.04 | 0.04 | 0.06 | 0.03 | 0.57 | 1.00 | - | 0.91 | 0.91 | 0.83 | 1.00 |
| **rs2283727** | 0.01 | 0.04 | 0.03 | 0.03 | 0.04 | 0.04 | 0.05 | 0.04 | 0.56 | 0.78 | 0.78 | - | 1.00 | 1.00 | 1.00 |
| **rs2283728** | 0.01 | 0.04 | 0.03 | 0.03 | 0.04 | 0.04 | 0.05 | 0.04 | 0.56 | 0.78 | 0.78 | 1.00 | - | 1.00 | 1.00 |
| **rs56220155** | 0.01 | 0.04 | 0.03 | 0.03 | 0.05 | 0.05 | 0.07 | 0.04 | 0.36 | 0.51 | 0.51 | 0.67 | 0.67 | - | 1.00 |
| **rs4824562** | 0.00 | 0.00 | 0.00 | 0.00 | 0.00 | 0.00 | 0.00 | 0.00 | 0.02 | 0.05 | 0.05 | 0.05 | 0.05 | 0.07 | - |
| **Mothers of Male Probands** | | | | | | | | | | | | | | | |
| **30bp-uVNTR** | - | 0.80 | 0.80 | 0.80 | 0.76 | 0.76 | 0.74 | 0.77 | 0.14 | 0.24 | 0.24 | 0.27 | 0.27 | 0.26 | 0.05 |
| **rs5906883** | 0.61 | - | 0.98 | 0.98 | 0.88 | 0.88 | 0.79 | 0.93 | 0.20 | 0.34 | 0.34 | 0.44 | 0.44 | 0.31 | 0.10 |
| **rs1465107** | 0.59 | 0.94 | - | 1.00 | 0.85 | 0.85 | 0.79 | 0.90 | 0.26 | 0.35 | 0.35 | 0.41 | 0.41 | 0.29 | 0.15 |
| **rs1465108** | 0.59 | 0.94 | 1.00 | - | 0.85 | 0.85 | 0.79 | 0.90 | 0.26 | 0.35 | 0.35 | 0.41 | 0.41 | 0.29 | 0.15 |
| **rs5905809** | 0.46 | 0.65 | 0.63 | 0.63 | - | 1.00 | 0.79 | 0.79 | 0.41 | 0.44 | 0.44 | 0.52 | 0.52 | 0.33 | 0.03 |
| **rs5906957** | 0.46 | 0.65 | 0.63 | 0.63 | 1.00 | - | 0.79 | 0.79 | 0.41 | 0.44 | 0.44 | 0.52 | 0.52 | 0.33 | 0.03 |
| **rs6323** | 0.52 | 0.62 | 0.60 | 0.60 | 0.52 | 0.52 | - | 0.80 | 0.42 | 0.46 | 0.46 | 0.41 | 0.41 | 0.38 | 0.08 |
| **rs1137070** | 0.52 | 0.80 | 0.77 | 0.77 | 0.58 | 0.58 | 0.58 | - | 0.34 | 0.42 | 0.42 | 0.50 | 0.50 | 0.36 | 0.14 |
| **rs3027440** | 0.00 | 0.00 | 0.01 | 0.01 | 0.03 | 0.03 | 0.02 | 0.02 | - | 0.89 | 0.89 | 0.76 | 0.76 | 0.74 | 0.54 |
| **rs6324** | 0.01 | 0.02 | 0.02 | 0.02 | 0.04 | 0.04 | 0.04 | 0.03 | 0.65 | - | 1.00 | 0.79 | 0.79 | 0.81 | 0.81 |
| **rs3027441** | 0.01 | 0.02 | 0.02 | 0.02 | 0.04 | 0.04 | 0.04 | 0.03 | 0.65 | 1.00 | - | 0.79 | 0.79 | 0.81 | 0.81 |
| **rs2283727** | 0.01 | 0.03 | 0.03 | 0.03 | 0.06 | 0.06 | 0.03 | 0.05 | 0.46 | 0.61 | 0.61 | - | 1.00 | 1.00 | 0.43 |
| **rs2283728** | 0.01 | 0.03 | 0.03 | 0.03 | 0.06 | 0.06 | 0.03 | 0.05 | 0.46 | 0.61 | 0.61 | 1.00 | - | 1.00 | 0.43 |
| **rs56220155** | 0.01 | 0.02 | 0.02 | 0.02 | 0.03 | 0.03 | 0.04 | 0.04 | 0.32 | 0.47 | 0.47 | 0.72 | 0.72 | - | 0.50 |
| **rs4824562** | 0.00 | 0.00 | 0.01 | 0.01 | 0.00 | 0.00 | 0.00 | 0.01 | 0.02 | 0.07 | 0.07 | 0.02 | 0.02 | 0.04 | - |
| **Female Probands** | | | | | | | | | | | | | | | |
| **30bp-uVNTR** | - | 0.59 | 0.62 | 0.62 | 0.74 | 0.74 | 0.52 | 0.59 | 0.11 | 0.11 | 0.11 | 0.02 | 0.02 | 0.07 | 0.25 |
| **rs5906883** | 0.32 | - | 1.00 | 1.00 | 0.86 | 0.86 | 0.48 | 0.56 | 0.17 | 0.38 | 0.38 | 0.31 | 0.31 | 0.13 | 0.31 |
| **rs1465107** | 0.38 | 0.91 | - | 1.00 | 0.87 | 0.87 | 0.51 | 0.59 | 0.10 | 0.46 | 0.46 | 0.25 | 0.25 | 0.19 | 0.37 |
| **rs1465108** | 0.38 | 0.91 | 1.00 | - | 0.87 | 0.87 | 0.51 | 0.59 | 0.10 | 0.46 | 0.46 | 0.25 | 0.25 | 0.19 | 0.37 |
| **rs5905809** | 0.33 | 0.41 | 0.46 | 0.46 | - | 1.00 | 1.00 | 0.62 | 0.37 | 0.71 | 0.71 | 0.51 | 0.51 | 0.10 | 0.11 |
| **rs5906957** | 0.33 | 0.41 | 0.46 | 0.46 | 1.00 | - | 1.00 | 0.62 | 0.37 | 0.71 | 0.71 | 0.51 | 0.51 | 0.10 | 0.11 |
| **rs6323** | 0.27 | 0.21 | 0.26 | 0.26 | 0.60 | 0.60 | - | 0.46 | 0.46 | 0.57 | 0.57 | 0.70 | 0.70 | 0.02 | 0.02 |
| **rs1137070** | 0.32 | 0.27 | 0.32 | 0.32 | 0.25 | 0.25 | 0.20 | - | 1.00 | 0.53 | 0.53 | 0.32 | 0.32 | 0.20 | 0.16 |
| **rs3027440** | 0.00 | 0.01 | 0.00 | 0.00 | 0.03 | 0.03 | 0.03 | 0.17 | - | 0.86 | 0.86 | 0.68 | 0.68 | 0.77 | 1.00 |
| **rs6324** | 0.00 | 0.02 | 0.03 | 0.03 | 0.14 | 0.14 | 0.05 | 0.05 | 0.74 | - | 1.00 | 0.84 | 0.84 | 0.77 | 1.00 |
| **rs3027441** | 0.00 | 0.02 | 0.03 | 0.03 | 0.14 | 0.14 | 0.05 | 0.05 | 0.74 | 1.00 | - | 0.84 | 0.84 | 0.77 | 1.00 |
| **rs2283727** | 0.00 | 0.01 | 0.01 | 0.01 | 0.09 | 0.09 | 0.10 | 0.02 | 0.36 | 0.55 | 0.55 | - | 1.00 | 1.00 | 1.00 |
| **rs2283728** | 0.00 | 0.01 | 0.01 | 0.01 | 0.09 | 0.09 | 0.10 | 0.02 | 0.36 | 0.55 | 0.55 | 1.00 | - | 1.00 | 1.00 |
| **rs56220155** | 0.00 | 0.01 | 0.03 | 0.03 | 0.00 | 0.00 | 0.00 | 0.02 | 0.25 | 0.25 | 0.25 | 0.54 | 0.54 | - | 1.00 |
| **rs4824562** | 0.01 | 0.01 | 0.03 | 0.03 | 0.00 | 0.00 | 0.00 | 0.00 | 0.06 | 0.06 | 0.06 | 0.08 | 0.08 | 0.16 | - |
| **Parents of Female Probands** | | | | | | | | | | | | | | | |
| **30bp-uVNTR** | - | 0.68 | 0.68 | 0.68 | 0.66 | 0.66 | 0.67 | 0.46 | 0.30 | 0.47 | 0.47 | 0.57 | 0.57 | 0.04 | 0.59 |
| **rs5906883** | 0.42 | - | 1.00 | 1.00 | 0.90 | 0.90 | 0.89 | 0.56 | 0.40 | 0.54 | 0.54 | 0.62 | 0.62 | 0.08 | 0.62 |
| **rs1465107** | 0.42 | 1.00 | - | 1.00 | 0.90 | 0.90 | 0.89 | 0.56 | 0.40 | 0.54 | 0.54 | 0.62 | 0.62 | 0.08 | 0.62 |
| **rs1465108** | 0.42 | 1.00 | 1.00 | - | 0.90 | 0.90 | 0.89 | 0.56 | 0.40 | 0.54 | 0.54 | 0.62 | 0.62 | 0.08 | 0.62 |
| **rs5905809** | 0.36 | 0.74 | 0.74 | 0.74 | - | 1.00 | 1.00 | 0.52 | 1.00 | 1.00 | 1.00 | 1.00 | 1.00 | 0.08 | 0.65 |
| **rs5906957** | 0.36 | 0.74 | 0.74 | 0.74 | 1.00 | - | 1.00 | 0.52 | 1.00 | 1.00 | 1.00 | 1.00 | 1.00 | 0.08 | 0.65 |
| **rs6323** | 0.41 | 0.65 | 0.65 | 0.65 | 0.75 | 0.75 | - | 0.53 | 1.00 | 1.00 | 1.00 | 1.00 | 1.00 | 0.11 | 1.00 |
| **rs1137070** | 0.21 | 0.28 | 0.28 | 0.28 | 0.23 | 0.23 | 0.26 | - | 1.00 | 0.47 | 0.47 | 0.57 | 0.57 | 0.06 | 1.00 |
| **rs3027440** | 0.01 | 0.02 | 0.02 | 0.02 | 0.18 | 0.18 | 0.13 | 0.15 | - | 1.00 | 1.00 | 0.84 | 0.84 | 0.81 | 1.00 |
| **rs6324** | 0.03 | 0.05 | 0.05 | 0.05 | 0.20 | 0.20 | 0.15 | 0.03 | 0.87 | - | 1.00 | 0.86 | 0.86 | 0.83 | 1.00 |
| **rs3027441** | 0.03 | 0.05 | 0.05 | 0.05 | 0.20 | 0.20 | 0.15 | 0.03 | 0.87 | 1.00 | - | 0.86 | 0.86 | 0.83 | 1.00 |
| **rs2283727** | 0.06 | 0.08 | 0.08 | 0.08 | 0.23 | 0.23 | 0.17 | 0.06 | 0.55 | 0.65 | 0.65 | - | 1.00 | 1.00 | 0.40 |
| **rs2283728** | 0.06 | 0.08 | 0.08 | 0.08 | 0.23 | 0.23 | 0.17 | 0.06 | 0.55 | 0.65 | 0.65 | 1.00 | - | 1.00 | 0.40 |
| **rs56220155** | 0.00 | 0.00 | 0.00 | 0.00 | 0.00 | 0.00 | 0.00 | 0.00 | 0.33 | 0.39 | 0.39 | 0.64 | 0.64 | - | 0.50 |
| **rs4824562** | 0.05 | 0.06 | 0.06 | 0.06 | 0.07 | 0.07 | 0.13 | 0.15 | 0.06 | 0.07 | 0.07 | 0.01 | 0.01 | 0.03 | - |
