# Supplementary material for: Pilot study indicate role of preferentially transmitted monoamine oxidase gene variants in behavioral problems of male ADHD probands
Source: BMC Med Genet. 2017 Oct 5;18:109. doi: 10.1186/s12881-017-0469-5 (PMC5629801; doi:10.1186/s12881-017-0469-5)
Supplement: Supplementary file 7 — Analysis of allelic association with CPRS-R ‘T scores’ for oppositional behavior and PACS scores for conduct problems in male ADHD probands. Description: The table summarizes statistical comparisons between the mean scores and MAO alleles. (PDF 23 kb) [file 12881_2017_469_MOESM7_ESM.pdf]

Additional file 7: Analysis of allelic association with CPRS-R 'T scores' for oppositional behavior and PACS scores for conduct problems in male ADHD probands

Genes	Variants	Alleles	CPRS-R 'T scores' for oppositional behavior		PACS scores for conduct problems	
			**Mean ± SE**	***p*-value**	**Mean ± SE**	***p*-value**
MAOA	30bp-uVNTR	*3R*	63.15 ± 1.65	0.28	16.35 ± 1.53	0.33
		4R	61.57 ± 2.00		17.50 ± 2.17	
	rs5906883	*A*	61.93 ± 1.62	0.23	16.40 ± 1.58	0.35
		C	63.91 ± 2.11		17.39 ± 2.03	
	rs1465107	*G*	65.16 ± 2.14	0.08	17.82 ± 2.08	0.27
		A	61.39 ± 1.59		16.18 ± 1.56	
	rs1465108	*A*	61.39 ± 1.59	0.08	16.18 ± 1.56	0.27
		G	65.16 ± 2.14		17.82 ± 2.08	
	rs5905809	*C*	62.80 ± 2.12	0.46	17.89 ± 2.06	0.26
		G	62.52 ± 1.62		16.15 ± 1.57	
	rs5906957	*A*	62.52 ± 1.62	0.46	16.15 ± 1.57	0.26
		G	62.80 ± 2.12		17.89 ± 2.06	
	rs6323	*T*	59.48 ± 2.51	0.08	18.30 ± 2.14	0.22
		G	63.67 ± 1.49		16.13 ± 1.52	
	rs1137070	*C*	64.30 ± 2.15	0.17	19.11 ± 1.95	0.09
		T	61.72 ± 1.60		15.53 ± 1.58	
MAOB	rs4824562	*A*	61.88 ± 1.43	0.13	17.42 ± 1.41	0.13
		G	65.52 ± 2.91		13.88 ± 2.62	
	rs56220155	*G*	63.06 ± 2.34	0.42	15.32 ± 2.31	0.25
		A	62.46 ± 1.54		17.25 ± 1.48	
	rs2283728	*T*	63.46 ± 2.57	0.37	14.67 ± 2.55	0.22
		C	62.39 ± 1.48		17.19 ± 1.41	
	rs2283727	*C*	62.39 ± 1.48	0.37	17.19 ± 1.41	0.22
		A	63.46 ± 2.57		14.67 ± 2.55	
	rs3027441	*C*	64.55 ± 2.60	0.21	16.00 ± 2.76	0.40
		T	62.08 ± 1.48		16.87 ± 1.39	
	rs6324	*C*	62.08 ± 1.48	0.21	16.87 ± 1.39	0.40
		T	64.55 ± 2.60		16.00 ± 2.76	
	rs3027440	*T*	62.37 ± 1.42	0.33	17.08 ± 1.39	0.25
		C	63.86 ± 3.07		14.67 ± 2.68	
