# Supplementary material for: Pilot study indicate role of preferentially transmitted monoamine oxidase gene variants in behavioral problems of male ADHD probands
Source: BMC Med Genet. 2017 Oct 5;18:109. doi: 10.1186/s12881-017-0469-5 (PMC5629801; doi:10.1186/s12881-017-0469-5)
Supplement: Supplementary file 8 — Analysis of allelic association with the status of age-of-onset of ADHD in the male probands. Description: The table summarizes statistical comparison of the early and/or late onset of disorder with MAO alleles. (PDF 25 kb) [file 12881_2017_469_MOESM8_ESM.pdf]

Additional file 8: Analysis of allelic association with the status of age-of-onset of ADHD in the male probands

Genes	Variants	Alleles	Early onset	Late onset	χ^2 (*p*-value)
MAOA	30bp-uVNTR	*3R*	0.71	0.63	1.35 (0.25)
		4R	0.29	0.37	
	rs5906883	*A*	0.69	0.64	0.3 (0.58)
		C	0.31	0.36	
	rs1465107	*G*	0.30	0.34	0.2 (0.65)
		A	0.70	0.66	
	rs1465108	*A*	0.70	0.66	0.2 (0.65)
		G	0.30	0.34	
	rs5905809	*C*	0.32	0.36	0.18 (0.67)
		G	0.68	0.64	
	rs5906957	*A*	0.68	0.64	0.18 (0.67)
		G	0.32	0.36	
	rs6323	*T*	0.24	0.29	0.44 (0.51)
		G	0.76	0.71	
	rs1137070	*C*	0.32	0.34	0.04 (0.84)
		T	0.68	0.66	
MAOB	rs4824562	*A*	0.82	0.80	0.06 (0.81)
		G	0.18	0.20	
	rs56220155	*G*	0.26	0.25	0.01 (0.92)
		A	0.74	0.75	
	rs2283728	*T*	0.18	0.21	0.26 (0.61)
		C	0.82	0.79	
	rs2283727	*C*	0.82	0.79	0.26 (0.61)
		A	0.18	0.21	
	rs3027441	*C*	0.19	0.21	0.13 (0.72)
		T	0.81	0.79	
	rs6324	*C*	0.81	0.79	0.13 (0.72)
		T	0.19	0.21	
	rs3027440	*T*	0.85	0.86	0.03 (0.87)
		C	0.15	0.14	
